# Thraustochytrid PUFA synthase ER domains form a stable heterodimer

**DOI:** 10.1016/j.yjsbx.2026.100150

**Published:** 2026-06-10

**Authors:** Nahuel Lofeudo, Gabriel Moncalian

**Affiliations:** Department of Molecular Biology, Institute of Biomedicine and Biotechnology of Cantabria (IBBTEC), University of Cantabria–CSIC, Santander, Spain

**Keywords:** Enoyl reductase, Polyketide synthase, Fatty acid synthase, Polyunsaturated fatty acid, DHA, EPA

## Abstract

Omega-3 polyunsaturated fatty acids (PUFAs) are essential nutrients for humans and are synthesized de novo by specialized enzymes known as PUFA synthases (Pfas). The domains of these enzymes are structurally related to those of mammalian or bacterial fatty acid synthases (FAS), as well as microbial polyketide synthases (PKS). Pfas are typically composed of three polypeptides in thraustochytrids and myxobacteria, or four in marine gammaproteobacteria. The enoyl-ACP reductase (ER) domain plays a key role in PUFA synthesis by catalyzing the reduction of carbon‑carbon double bonds during modification reactions. In gammaproteobacteria, a single ER domain is present as a standalone protein (PfaD) within the megasynthase. However, in thraustochytrids ER domains are found in both PfaB (ERb) and PfaC (ERc), although their specific functional roles remain unclear. Previous studies have shown that ER domains act as homodimers in Pfas, FAS, and PKS systems. Here, we investigate the PUFA synthase from the thraustochytrid *Schizochytrium* sp. and demonstrate that ERb and ERc interact to form a heterodimer, as confirmed by size-exclusion chromatography with multi-angle light scattering (SEC-MALS) and by a crystal structure solved at 2.2 Å resolution with bound flavin mononucleotide (FMN). These findings indicate that ER domains may facilitate dimerization between PfaB and PfaC. Furthermore, molecular docking and structural alignments support a ping-pong mechanism involving FMN and NADH for ERb and ERc activity. To our knowledge, this is the first reported crystal structure of a PUFA synthase ER-domain complex from thraustochytrids, providing new insights into the mechanism of action of these enzymes.

## Highlights

• First crystal structure of a thraustochytrid PUFA synthase ER-domain complex.
• scERb and scERc form a stable FMN-bound heterodimer.
• In vitro*,* scERb is unstable and requires scERc for folding and FMN binding.
• Docking analyses support a ping-pong mechanism for scERbc catalysis.

## Graphical abstract

**Figure:**
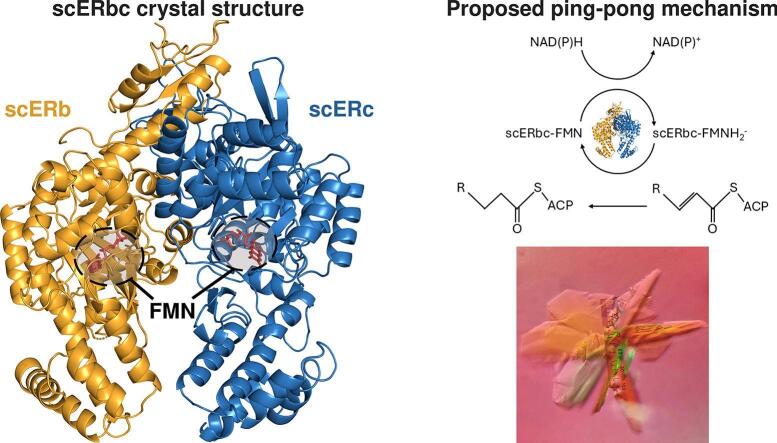

## 1 Introduction

**Fig. 1:**
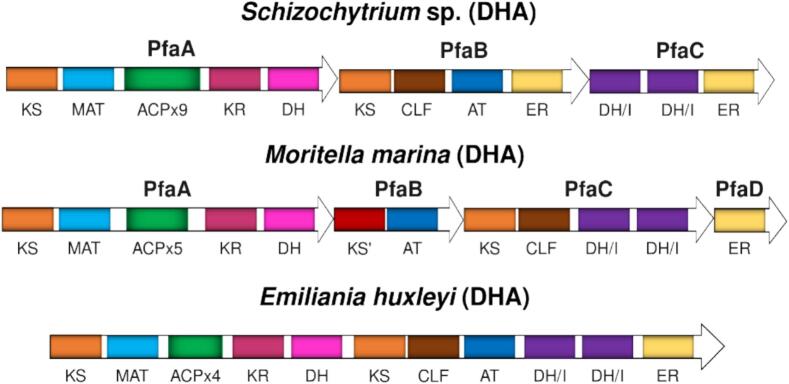
Domain organizations of *M. marina*, *Schizochytrium* sp. and *E. huxleyi* PUFA synthases. KS, ketosynthase; MAT, malonyl-CoA acyltransferase; ACP, acyl carrier protein; KR, ketoreductase; DH, dehydratase; AT, acyltransferase; CLF, chain length factor; and ER, enoyl reductase.

Omega-3 polyunsaturated fatty acids (PUFAs), such as docosahexaenoic acid (DHA, 22:6ω3) and eicosapentaenoic acid (EPA, 20:5ω3), are essential for human health (Swanson et al., 2012). However, current production, derived mainly from fish oil, is unsustainable due to overfishing, climate change, and increasing global demand. Estimates predict a shortfall of approximately 1 million tonnes by 2050, potentially affecting up to 90% of the global population (Jovanovic et al., 2021). Therefore, alternative and sustainable sources are urgently needed. In this context, marine microorganisms such as thraustochytrids and gammaproteobacteria produce PUFAs de novo via an anaerobic pathway mediated by PUFA synthases (Pfas). These enzymes are structurally and functionally related to type I fatty acid synthases (FAS) and polyketide synthases (PKS) (Lippmeier et al., 2009; Meesapyodsuk and Qiu, 2016; Metz et al., 2001; Zhao and Qiu, 2018). Pfas are large, multienzyme complexes composed of three multidomain polypeptides in thraustochytrids and myxobacteria, or four in gammaproteobacteria. They are encoded by gene clusters organized as *pfaA-C* or *pfaA-D*, respectively (Shulse and Allen, 2011). These complexes contain condensing (ketosynthase [KS], acyltransferase [AT]) and modifying (ketoreductase [KR], dehydratase [DH], enoyl-reductase [ER]) domains. They also comprise multiple tandem acyl carrier protein (ACP) domains, typically 5–9 copies within PfaA. Their number correlates with PUFA productivity (Hayashi et al., 2016; Jiang et al., 2008) (Fig. 1).

The thraustochytrid *Schizochytrium* sp. is widely used for commercial DHA production, with yields reaching up to 53% of total fatty acids (Yue et al., 2019). Typically, Pfa systems use malonyl-CoA as a starter unit and catalyze PUFA biosynthesis iteratively (Santín and Moncalián, 2018). A recent study by Ogata et al. (2025) in *Schizochytrium* sp. showed that PfaA-KS-MAT efficiently elongates the acyl chain to produce a C8-ACP intermediate. Subsequent elongation to C10-ACP is catalyzed by PfaB-KS-CLF, after which PfaA-KS-MAT further elongates the intermediate to C14-ACP. In the final stages of the pathway, the growing acyl chain is transferred multiple times between PfaA and PfaB, where the final elongation and termination steps occur, ultimately yielding DHA. This process mirrors PUFA biosynthesis in gammaproteobacteria, where PfaA-KS-MAT and PfaC-KS-CLF exhibit similar substrate specificity and catalytic activity (Hayashi et al., 2019; Santín et al., 2020). Another recent study (Lofeudo et al., 2026) reported that the AT domain in PfaB from gammaproteobacterial PUFA synthases exhibits acyltransferase activity, with substrate specificity distinct from that of PfaA-KS-MAT. The same study further showed that PfaB determines the final PUFA product. Furthermore, PfaB-AT domains from both bacterial and thraustochytrid PUFA synthases have been reported to display hydrolase activity against long-chain substrates (>C18) (Almendáriz-Palacios et al., 2021; Hayashi et al., 2020), contributing to product off-loading.

Modifying domains, in contrast, reduce intermediates to yield fully or partially saturated fatty acids. Notably, the KR and DH domains generate a double-bond intermediate, which is subsequently reduced by the ER domain using NADH or NADPH during the last step of modification reactions. ER domains exhibit broad structural diversity, and three superfamilies have been described: short-chain dehydrogenase/reductase (SDR), triose-phosphate isomerase (TIM) barrel, and medium-chain dehydrogenase/reductase (MDR) (Massengo-Tiassé and Cronan, 2009). Notably, gammaproteobacterial PUFA synthases contain a single TIM-barrel ER domain in PfaD (Zhang et al., 2021); a fold first identified in FabK from the bacterial type II FAS system (Saito et al., 2008). This architecture is non-canonical, as most bacterial ER FAS enzymes adopt the Rossmann fold of the SDR superfamily. In contrast, thraustochytrid PUFA synthases possess two ER domains in PfaB (ERb) and in PfaC (ERc) (Fig. 1), which share ∼50% sequence identity with gammaproteobacterial PfaD proteins (Metz et al., 2001). TIM-barrel ERs require dimerization for activity and bind FMN as a permanent cofactor. In addition, reducing cofactors NADH or NADPH are transiently bound during ER catalysis (Bukhari et al., 2014; Ha et al., 2017; Saito et al., 2008; Zhang et al., 2021).

The structure, function, and assembly of ERb and ERc in thraustochytrids remain uncharacterized. To address this knowledge gap, we investigated the interaction, oligomerization, and stability of the ER domains in *Schizochytrium* sp. ATCC 20888. Consistent with other eukaryotic PUFA synthases, this organism contains two ER domains (scERb and scERc). We hypothesized that these ER domains form a functional heterodimer that mediates the interaction between PfaB and PfaC. Here, we report the 2.2 Å crystal structure of the scERb–scERc complex (PDB 9SDD), with FMN bound in both domains. Additionally, we present docking studies of NAD^+^, the substrate crotonyl-CoA, ACP, and the inhibitor TUI. These findings provide the first crystal structure of a thraustochytrid PUFA synthase and significantly advance our understanding of its architecture and assembly, offering insights that may support the rational engineering of improved microbial PUFA production.

## 2 Results

### 2.1 Phylogenetic comparison of ER domains in eukaryotic and prokaryotic PUFA synthases

**Fig. 2:**
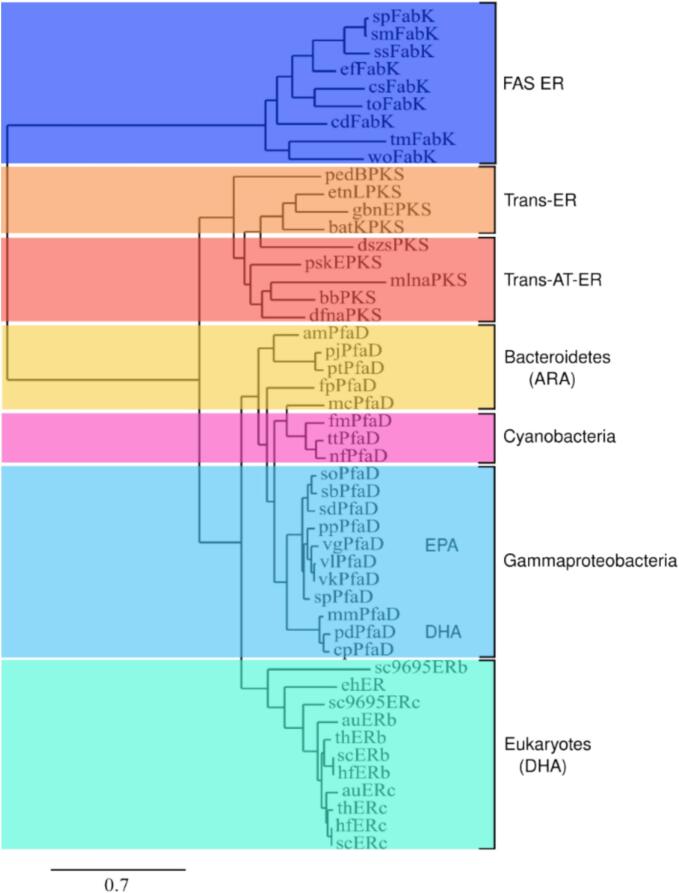
Phylogenetic tree and distribution of TIM-barrel ER domains. The tree is drawn to scale, with branch lengths representing the number of amino acid substitutions per site. Main groups are indicated: FabK, trans-ER PKS, trans-ER-AT PKS, PfaD, ERb, and ERc. Details of the sequences used are provided in Table S1.

As described in the introduction, eukaryotic PUFA synthases contain duplicated ER domains (ERb and ERc). Like other ER domains from FAS and PKS, ERb and ERc belong to the TIM-barrel superfamily. To investigate the evolutionary relationships and potential roles of the putative ER domains in eukaryotic PUFA synthases, we first compared their sequence and organization with other TIM-barrel ER domains. Phylogenetic analyses of selected ER domains from these synthases reveal that, although these enzymes belong to the same superfamily, FAS FabK is only distantly related to both PKS ER domains and those of PUFA synthases (PfaD, ERb, and ERc) (Fig. 2). Consistently, FabK proteins share only ∼20% identity with PKS or PUFA ER domains (Fig. S1).

Previous studies reported that ER domains from trans-AT PKS systems (PKS-ERs) are evolutionarily related to ER PfaD proteins from prokaryotic PUFA synthases (Bukhari et al., 2014), a relationship that is also reflected in our phylogenetic analyses. Most identified PKS-ERs are associated with one or two AT domains, forming trans-acting AT-ER proteins, although some function as standalone trans-acting enzymes (e.g., PedB, EtnL, GbnE, BatK) (Piel, 2010). Notably, both types of PKS ER domains share ∼40% identity with PfaD, ERb, and ERc (Fig. S1) and form separate clades in the phylogenetic tree (Fig. 2).

Bacteroidetes species that predominantly synthesize omega-6 arachidonic acid (e.g., *Aureispira marina*, *Flammeovirga pacifica*, *Psychroserpens jangbogonensis*, *Psychroflexus torquis*) harbor PfaD proteins that are evolutionarily distant from those of EPA- and DHA-producing gammaproteobacteria. In contrast, cyanobacteria such as *Nostoc flagelliforme*, *Tolypothrix tenuis*, and *Fischerella major* possess ER domains closely related to gammaproteobacterial PfaDs (Shulse and Allen, 2011) (Fig. 2).

Eukaryotic ER domains from thraustochytrids cluster closely with prokaryotic PfaD proteins, suggesting a shared evolutionary origin. Within thraustochytrids, ERb appears to be the earliest-diverging ER domain in the PUFA synthase. Notably, *Schizochytrium* sp. ATCC 9695 forms a distinct clade separate from *Schizochytrium* sp. ATCC 20888 (Fig. 2). The ERb domain from ATCC 9695 (scERb9695) shows an average of ∼40% identity to bacterial PfaD proteins and ∼50% identity to other thraustochytrid ER domains (Fig. S1). In contrast, its ERc domain (scERc9695) shares 74% identity with ERb and 77% with ERc homologs, supporting the hypothesis that ERc arose later through duplication of ERb (Fig. S1).

Remarkably, the ER domain of the haptophyte *Emiliania huxleyi* (ehER) appears to have evolved from scERb9695, possibly via lateral gene transfer. Although *E. huxleyi* synthesizes DHA, the architecture of its PUFA synthase differs substantially from that of thraustochytrids (Morabito et al., 2019), resembling the single-polypeptide organization of mammalian FAS (Maier et al., 2008) (Fig. 1). The ehER domain shares 48% identity with scERb9695 and approximately 60% identity with other ERb and ERc domains (Fig. S1).

Across thraustochytrids, scERb shares 80–85% sequence identity with ERb domains from *Thraustochytrium* sp. and *Aurantiochytrium* sp., whereas scERc shares 85–94% identity with ERc homologs in these species. Overall, ERb and ERc domains are highly similar to each other, with 75–80% identity in cross-comparisons. In contrast, prokaryotic PfaDs share only ⁓50% identity with thraustochytrid ERb and ERc domains, consistent with previous reports (Metz et al., 2001).

PfaD proteins contain a characteristic ∼80 amino acid N-terminal extension (Bukhari et al., 2014), which is absent in FAS or PKS ER domains (Fig. S2). Thraustochytrid ER domains retain this feature, in agreement with their phylogenetic proximity to PfaDs. In addition, conserved insertions previously described for PfaD and PKS-ERs relative to FabK (Bukhari et al., 2014) are also present in scERb and scERc. These features likely explain the formation of distinct clades in the phylogenetic tree.

### 2.2 Expression, oligomerization, and stability of ERb and ERc domains

We further characterized the eukaryotic PUFA synthase ER domains by studying in vitro the isolated ER domains from *Schizochytrium* sp. ATCC 20888, corresponding to the C-terminal regions of PfaB (scERb, residues 1538–2059) and PfaC (scERc, residues 982–1502). Heterologous expression in *Escherichia coli* used various vector constructs and fusion tags to optimize solubility and to enable purification.

scERb was initially expressed from a pET29c construct containing a C-terminal His-tag (predicted molecular weight [MW]: 57.19 kDa). However, under these conditions the protein was insoluble and could not be purified. To improve solubility, scERb was cloned into the pMAL-c2x vector with an MBP fusion tag (MBP MW: 42.66 kDa; resulting construct MW: 99.85 kDa). The fusion protein was further analyzed by size-exclusion chromatography (SEC) as described in Materials and Methods. As shown in Fig. S3A, the SEC profile of scERb-MBP displayed a major UV 280 nm peak at 8.2 mL, corresponding to the void volume, indicating aggregation. SDS-PAGE analysis confirmed the presence of aggregated scERb in these fractions (Fig. S3B). Notably, the purified protein appeared transparent, suggesting the absence of the characteristic yellow flavin mononucleotide (FMN) cofactor. Attempts to remove the MBP tag by Factor Xa digestion resulted in protein precipitation (Fig. S3C—D).

In contrast, the scERc domain (predicted MW: 59.06 kDa) expressed from a pET29c His-tag construct was highly soluble after affinity purification. Unlike scERb, scERc-containing fractions appeared yellow, indicating bound FMN cofactor. SEC analysis revealed an 8.2 mL peak and another major peak corresponding to an apparent MW of ∼100 kDa, consistent with a predominantly dimeric species. A shoulder on the main peak indicated a minor monomeric population (59.06 kDa) in equilibrium with the dimer (Fig. S4A). SDS-PAGE confirmed the presence of scERc in both peaks (Fig. S4B). Size-exclusion chromatography with multi-angle light scattering (SEC-MALS) analysis further showed that scERc predominantly exists as a homodimer (102.3 kDa), with a minor monomeric fraction (63.18 kDa) and a small peak likely corresponding to degradation products (34.55 kDa) (Fig. S5A). These observations are consistent with previous reports describing TIM-barrel ER domains as dimeric in solution (Bukhari et al., 2014; Ha et al., 2017; Saito et al., 2008; Zhang et al., 2021).

**Fig. 3:**
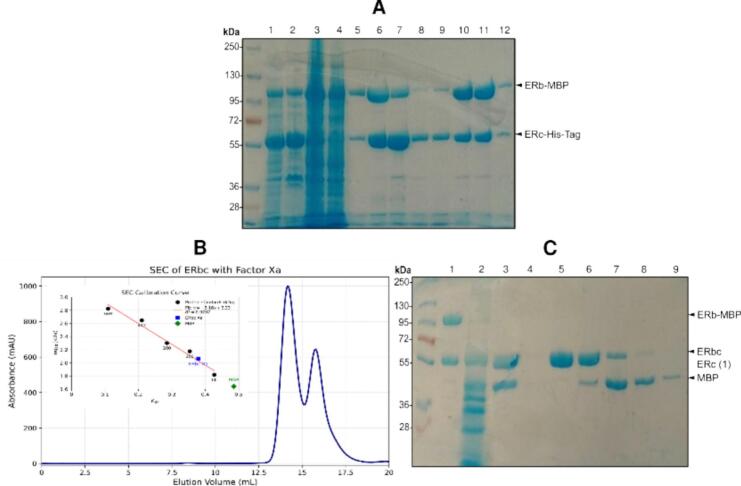
(A) SDS-PAGE analysis (8% acrylamide) of scERbc affinity chromatography purification. Lane 1, overexpression at 18 °C overnight, OD₆₀₀ = 0.6; lane 2, insoluble fraction; lane 3, soluble fraction; lane 4, flow-through from HisTrap (5 mL); lanes 5–8, elution fractions from the imidazole gradient; lane 9, flow-through from MBPTrap (5 mL); lanes 10–12, elution fractions from the maltose gradient. (B) Size-exclusion chromatography (SEC) elution profile of scERbc incubated with Factor Xa and calibration curve. Black circles indicate protein standards (kDa) with labels, the red line represents the linear regression fit, and the corresponding R^2^ is indicated. The blue square shows scERbc after Factor Xa treatment, and the green diamond corresponds to MBP. (C) SDS-PAGE analysis (8% acrylamide) of scERbc SEC purification. Lane 1, scERbc collected from affinity chromatography before Factor Xa treatment; lane 2, pellet after Factor Xa incubation; lane 3, soluble fraction after Factor Xa incubation; lanes 4–6, fractions from peak at 14.18 mL; lanes 7–9, fractions from peak at 15.77 mL. (For interpretation of the references to colour in this figure legend, the reader is referred to the web version of this article.)

Potential interactions between the two scER domains were assessed by co-expression in *E. coli* BL21(DE3). Sequential affinity purification using Ni-NTA and MBP Trap columns yielded a soluble scERb–scERc complex (scERbc), indicating the formation of a stable heterodimer with bound FMN (Fig. 3A). The SEC profile of scERbc displayed two peaks: one at 8.4 mL (void volume) and a second at 13.13 mL (Fig. S6A). SDS-PAGE analysis confirmed the presence of both scERb and scERc in the latter peak, supporting complex formation (Fig. S6B). The estimated MW of the complex (167 kDa) was slightly higher than the theoretical value (157.04 kDa) but remains consistent with heterodimer formation.

Following Factor Xa cleavage of the MBP tag (expected MW: 116.25 kDa), the complex eluted at ∼100 kDa, consistent with a compact heterodimeric species, along with a minor fraction that may correspond to partially dissociated monomers (Fig. 3B). SDS-PAGE confirmed the presence of both scERb and scERc (Fig. 3C). SEC-MALS analysis further supported the formation of a stable scERbc heterodimer, with a calculated MW of ∼106 kDa, as well as a minor peak corresponding to cleaved MBP (40.92 kDa) (Fig. S5B).

Thermal stability was assessed using Sypro Orange-based thermal shift assays. MBP-scERb did not produce a characteristic melting curve (*n* = 8). Instead, fluorescence decreased over time, indicating instability in solution (Fig. S6). In contrast, no significant difference was observed between MBP-tagged scERbc (Tm = 39.21 ± 1.18 °C, *n* = 12) and scERc (Tm = 37.88 ± 2.02 °C, n = 12), suggesting comparable stability. These relatively low melting temperatures are consistent with the marine origin of *Schizochytrium* sp., where enzymes are expected to be cold-adapted (Fig. S7).

### 2.3 scERbc overall crystal structure

As a preliminary step, the scERc domain was successfully crystallized and its structure refined to a resolution of 3.8 Å. At this resolution, amino acid side chains could not be clearly resolved, precluding detailed structural interpretation. The unit cell belongs to space group P3_1_2_1_, with the asymmetric unit containing a dimer. This observation is consistent with the dimeric state detected by SEC-MALS and with previous structural studies of TIM-barrel ER domains. Due to the limited resolution obtained, this crystal structure was not deposited in the PDB database.

In addition, the structure of the scERbc heterodimer was solved and deposited in the Protein Data Bank under the ID 9SDD. The structure was determined by molecular replacement using AlphaFold3-predicted models as search templates and refined to 2.2 Å resolution (Table 1).

**Table 1:** Data collection and refinement statistics (molecular replacement).

scERbc (PDB 9SDD)	
Data collection	
Space group	I1_2_1
Unit cell, α = γ = 90 β = 101.108	117.968, 79.108, 119.447
Wavelength (Å)	0.97929
Resolution range (Å)	45.91–2.2
Reflections total/unique	106,904 / 54,632
% completeness (last shell)	99.4 (94.4)
Multiplicity (last shell)	1.96 (1.9)
Average *I/σ* (last shell)	17.2 (3.8)
Rmerge (last shell)	0.017 (0.145)
CC1/2 (last shell)	0.999 (0.955)

Refinement	
R_work_/R_free_	0.1868 / 0.2302
Protein atoms	8212
Root mean square deviations	
Bond lengths (Å)	0.007
Bond angles (°)	0.8

Ramachandran plot	
Favored	98.16
Allowed	1.74
Outlier	0.1

**Fig. 4:**
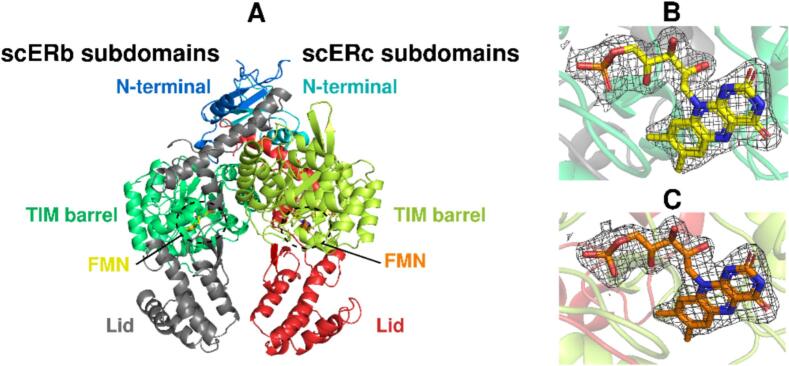
(A) Overall structure of ERbc with bound FMN (stick representation; yellow in scERb; orange in scERc). The ER domains (cartoon representation) comprise three subdomains: N-terminal (blue in scERb; cyan in scERc), TIM barrel (green in scERb; lime in scERc) and lid (gray in scERb; red in scERc). (B) Close-up view of the FMN 2F_o_ – F_c_ electron density map in scERb, shown as a black isomesh contoured at 1.0 σ. (C) FMN 2F_o_ – F_c_ electron density map in scERc, displayed as a black isomesh contoured at 1.0 σ. (For interpretation of the references to colour in this figure legend, the reader is referred to the web version of this article.)

The scERbc structure is organized into three distinct subdomains. The N-terminal subdomain appears to be exclusive to PUFA synthase ER domains (Bukhari et al., 2014), and similar to those of *Shewanella oneidensis* PfaD (soPfaD; PDB 4YX6; RMSD 1.236 Å), or *Shewanella piezotolerans* PfaD (spPfaD; PDB 6LKC; RMSD 1.758 Å) (Zhang et al., 2021). In scERbc, this subdomain spans residues 1538–1590 in scERb and 985–1036 in scERc (Fig. 4A). Structural differences are evident: scERb contains 2 β-strands, 1 α-helix, and 1 η-helix, whereas scERc includes an additional β-strand. This variation likely arises from differences in the cloning constructs used. Similarly, the N-terminal subdomain of soPfaD comprises 3 β-strands, 1 α-helix, and 1 η-helix. In contrast, the equivalent region in spPfaD contains 1 α-helix, 6 β-strands, and 3 η-helices (Zhang et al., 2021). The asymmetric unit contains a heterodimer, with FMN bound in both scERb and scERc (Fig. 4B-C).

The central TIM-barrel core subdomain of scERb (residues 1591–1862) and scERc (residues 1037–1308) comprises 8 α-helices and 7 β-strands. Both domains deviate from the canonical TIM-barrel fold, which typically contains 8 β-strands. In contrast, other ERs such as *Streptococcus pneumoniae* FabK (spFabK; PDB 2Z6I; RMSD 2.869 Å) (Saito et al., 2008), spPfaD, soPfaD, the PKS-ERs DfnA (PDB 4CW5; RMSD 3.27 Å) and MlnA (PDB 4Z38; RMSD 1.175 Å) (Bukhari et al., 2014), display the canonical 8 β-strands in this region (Fig. S8). Despite this difference, the overall fold is conserved and closely resembles that of other known ER domains. In addition to the core, both domains contain 2 extra antiparallel β-strands (residues 1705–1715 in scERb and 1151–1161 in scERc) flanked by 3 α-helices. These features are also observed in PfaD proteins and PKS ER domains (Fig. S2).

The lid subdomain comprises residues 1863–2056 in scERb and 1309–1502 in scERc, and contains 2 β-strands, 2 η-helices, and 9 α-helices. Notably, scERb possesses an additional α-helix (residues 2040–2048) absent in scERc and other characterized ER domains (Fig. S9). Moreover, scERb and other ERb domains from related thraustochytrids (e.g. *Auriantiochytrium* sp., *Thraustochytrium* sp.) harbor a three-residue insertion (scERb residues 2045–2047), forming an x[*LV*]x motif absent in ERc domains. Consistently, AlphaFold3 models predict an α-helix at the equivalent position in these related ERb domains. Despite the variations observed in the N-terminal and lid subdomains, the overall fold is highly similar between scERb and scERc. The root-mean-square deviation (RMSD) between the two domains is 0.617 Å, indicating strong structural conservation.

### 2.4 scERbc interface analysis

We next focused on the characterization of the scERbc interface to investigate the basis of heterodimer formation and to compare it with previously resolved TIM-barrel ER homodimers.

PISA analysis (Krissinel and Henrick, 2007) revealed an extensive scERbc interface (2366.7 Å^2^), containing 27 hydrogen bonds and 11 salt bridges. Despite its large size and extensive network of intermolecular contacts, the solvation free energy gain upon interface formation was relatively low (ΔiG = −11.1 kcal/mol). Moreover, this interface displayed a high ΔiG *p*-value (0.423) (Table 2).

**Table 2:** Interface analysis of selected TIM-barrel ER-domain dimers. The table summarizes intermolecular contacts, including hydrogen bonds, salt bridges, hydrophobic interactions, water bridges, and π-stacking interactions; together with the interface area, the solvation free energy gain upon interface formation (ΔiG), and the associated ΔiG *p*-value. Values were obtained from PISA and PLIP analyses. Structures correspond to experimentally determined assemblies, or to AlphaFold3-predicted models, as indicated. AlphaFold3 models were generated using the same scERb and scERc construct boundaries employed for recombinant expression and purification; n.d., not determined.

Protein	H-bonds	Salt bridges	Hydrophobic interactions	Water bridges	Interface area (Å^2^)	ΔiG (kcal/mol)	ΔiG p-value
scERbc (PDB 9SDD)	27	11	17	13	2365.4	−11.1	0.423
scERc (AlphaFold3)	32	20	18	n.d.	2647.5	−11.0	0.506
scERb (AlphaFold3)	18	4	16	n.d.	2226.8	−13.8	0.290
soPfaD (PDB 4YX6)	8	5	10	15	1760.7	−27.3	0.042
spPfaD (PDB 6LKC)	11	9	15	21	1968.6	−28.3	0.017
spFabK (PDB 2Z6I)	18	4	34	16	2358.8	−42.9	0.002
tmFabK (PDB 5GVH)	30	12	40	n.d.	2211.8	−26.1	0.047

To contextualize these observations, scERbc was compared with a dimeric model of scERc predicted by AlphaFold3. The predicted homodimer exhibited a larger interface area (2647.5 Å^2^), comparable numbers of hydrogen bonds (32) and nearly twice the number of salt bridges (20). Nevertheless, the calculated solvation free energy gain was very similar to that of scERbc (ΔiG = −11.0 kcal/mol), and the ΔiG p-value was likewise elevated (0.506). Further analysis with PLIP (Schake et al., 2025) identified a comparable number of hydrophobic interactions in scERbc and in the predicted scERc model (17 and 18, respectively), while scERbc contained 13 water bridges (Table 2).

An AlphaFold3 model of the scERb homodimer was also analyzed for comparison. Relative to scERbc and the modeled scERc homodimer, the predicted scERb interface displayed fewer hydrogen bonds (18) and a smaller interface area (2226.8 Å^2^), while retaining similar hydrophobic interaction counts (16) and comparable overall interface energetics (Table 2).

In contrast, the experimentally resolved PfaD proteins, soPfaD and spPfaD, display smaller interface areas (1760.7 and 1968.6 Å^2^, respectively), substantially fewer hydrogen bonds, and reduced numbers of salt bridges and hydrophobic interactions. Despite this, both proteins exhibited more favorable ΔiG values than either scERbc or the predicted scERc homodimer (Table 2).

The analyzed FabK-ERs (spFabK and tmFabK) showed interface areas comparable to that of scERbc (2358.8 and 2211.8 Å^2^, respectively). These proteins displayed substantially higher numbers of hydrophobic interactions (34 and 40, respectively). Although tmFabK and scERbc displayed similar numbers of polar contacts, FabK dimers exhibited markedly more favorable ΔiG values. This difference may partly reflect the larger hydrophobic contribution in FabK interfaces (Table 2). Water bridges were not determined for tmFabK, as the reported asymmetric unit was composed of a monomer (Ha et al., 2017).

Together, these results indicate that the dimerization modes of scERbc and the modeled scERc differ from those observed in the TIM-barrel ER domains analyzed. Both interfaces are enriched in polar contacts, while the scERbc interface additionally contains multiple water-mediated interactions. The predicted scERc homodimer exhibited a similar energetic profile to scERbc.

Having established the overall architecture of the scERbc heterodimer, attention was then turned to the conserved features of the active site that may underlie enzymatic specificity or reactivity.

### 2.5 Conservation of catalytic residues in the scERbc active site and comparison with related enzymes

A conserved histidine has been proposed as the catalytic residue in both FabK (Ha et al., 2017; Saito et al., 2008) and PfaD (Zhang et al., 2021). This residue is also conserved in ERb (H1776 in scERb) and ERc homologs (H1222 in scERc).

Among TIM-barrel enzymes, 2-nitropropane monooxygenases (2-NMOs) exhibit the highest sequence similarity to FabK (∼30%), with lower identity to PKS ERs, PfaD, ERb, and ERc (∼18%). In these enzymes, a histidine at an equivalent position has also been proposed as catalytic (Jun et al., 2006; Salvi et al., 2014). Motifs I-III characteristic of this enzyme family are retained in ER domains.

**Fig. 5:**
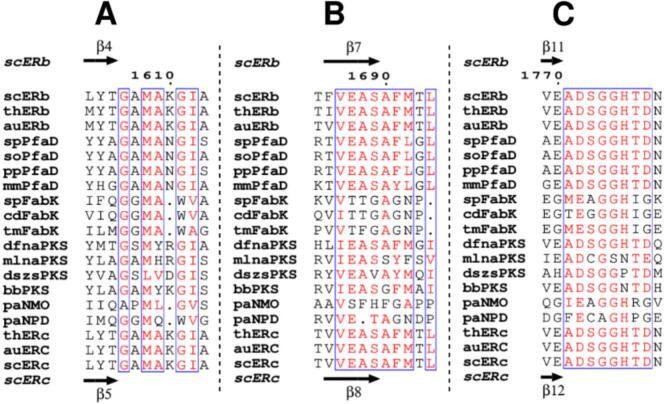
Structure-based multiple sequence alignment of ERbc. Numbering corresponds to ERb. Sequence homology is marked in red; sequence identity is presented with white letters on a red background. ERb and ERc secondary structure features (arrows for β-strands, and coils for α-helices) are indicated at the top and bottom, respectively. (A) Motif I. (B) Motif II. (C) Motif III. The UniProt accession numbers of the proteins used in the alignment are as follows: scERb, *Schizochytrium* sp. (Q94FB7); thERb, *Thraustochytrium* sp. (A0A1B3PEI8); auERb, *Auriantiochytrium* sp. (A0A7H0U711); spPfaD, *Shewanella piezotolerans* (B8CQB6); soPfaD, *S. oneidensis* (Q8EGK4); ppPfaD, *Photobacterium profundum* (Q93CG5); mmPfaD, *Moritella marina* (A0A5J6WHZ7); spFabK, *Streptococcus pneumoniae* (A0A0H2UNJ5); cdFabK, *Clostridioides difficile,* (A0A0H3N0T0); tmFabK, *Thermotoga maritima,* (Q9WZQ7); dfnaPKS, *Bacillus velezensis* (A7Z6E3); mlnaPKS, *B. velezensis* (A7Z470); dszsPKS, *Sorangium cellulosum* (Q4U443); bbPKS, *Brevibacillus brevis* (C0ZGR0); paNMO, *Pseudomonas aeruginosa*, (Q9HWH9); paNPD, *P. aeruginosa* (Q9I4V0); thERc, *Thraustochytrium* sp. (A0A1B3PEI9); auERc, *Auriantiochytrium* sp. (A0A7H0U712); scERc, *Schizochytrium* sp. (Q94FB6). The alignment was plotted with ENDscript. (For interpretation of the references to colour in this figure legend, the reader is referred to the web version of this article.)

Motif I (AP**M**x**G**) corresponds in ERb and ERc domains to the sequence G^1606^ A**M**AK**G** (notation refers to scERb). Within this motif, the methionine residue is highly conserved and has been reported to interact with FMN. Adjacent to this methionine, 2-NMOs feature a variable residue followed by a highly conserved glycine, which is substituted by tryptophan in FabK. Notably, ER domains contain an insertion between the X and M residues that is absent in both FabK and 2-NMOs (Fig. 5A).

Motif II ([VI]SF**H**F[*GN*]x), corresponding to V^1685^EA**S**AFM in ERb and ERc domains, is poorly conserved. In 2-NMOs it includes a histidine near FMN, whereas in ER domains the equivalent residue is replaced by serine, and in FabK by glycine (Fig. 5B).

Motif III (x**E**AGG**H**) is conserved in ER domains and corresponds to the sequence A^1771^DSGG**H** in ERb and ERc. This region includes the proposed catalytic histidine (Fig. 5C).

We next examined how these conserved residues, particularly those within the TIM-barrel, interact with the FMN cofactor to support catalytic activity in scERb and scERc.

### 2.6 scERbc interactions with cofactors

#### 2.6.1 FMN interaction

All TIM-barrel ER domains, including scERb and scERc, use FMN as a cofactor, with NADH or NADPH serving as electron donors. FMN is non-covalently accommodated within the TIM-barrel subdomain through a surface-accessible cleft (Fig. 4).

**Fig. 6:**
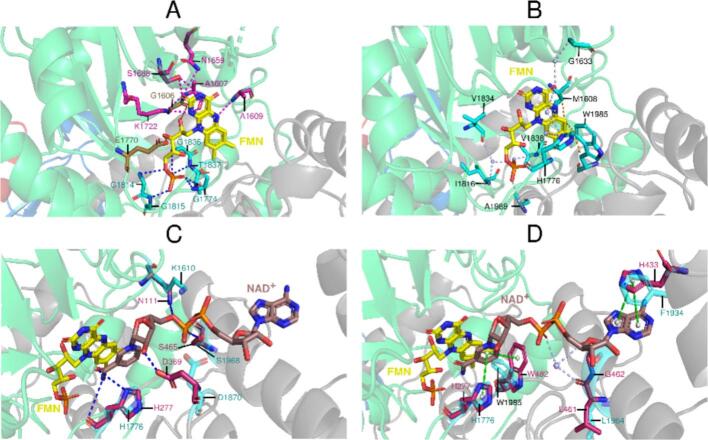
(A) Hydrogen-bonding interactions of FMN (yellow sticks) within scERb (cartoon representation). Hydrogen bonds are depicted as dashed lines: interacting residues are shown as sticks; magenta for interactions with the isoalloxazine ring, brown for the ribitol moiety, and cyan for the phosphate group. (B) Hydrophobic contacts (orange dashed lines) and water-mediated interactions (light-blue dashed lines) of FMN in scERb (residues are shown as cyan sticks). Water molecules are represented as light-blue spheres. (C) Predicted interactions of NAD^+^ (brown sticks) with scERbc (cartoon representation), derived from structural alignment with the soPfaD-NAD^+^ crystal structure (PDB 4Z9R). Residues from the soPfaD–NAD^+^ complex forming hydrogen bonds (blue dashed lines) are depicted as dark magenta sticks, along with their corresponding residues in scERb (cyan sticks). (D) Water bridges (light-blue dashed lines) and π-stacking interactions (green dashed lines). soPfaD residues are shown in dark magenta sticks and scERb residues are shown in cyan sticks. Water molecules are shown as light-blue spheres. (For interpretation of the references to colour in this figure legend, the reader is referred to the web version of this article.)

Given the low RMSD between scERb and scERc, and their high sequence identity, scERb was selected as a model to study the interactions identified by PISA (Krissinel and Henrick, 2007) and the PLIP server (Schake et al., 2025). The FMN cofactor forms hydrogen bonds with several residues within the TIM-barrel subdomain. In scERb, the isoalloxazine ring interacts with A1607 and A1609 from motif I, N1659 and S1688 from motif II, as well as K1722 and E1770. The ribitol moiety establishes contacts with G1606 and E1770, while the phosphate group forms hydrogen bonds with G1814, G1815, G1774, G1836, and T1837 (Fig. 6A).

The catalytic H1776 forms two water bridges with FMN via its ND1 atom (Fig. 6B). This interaction is also observed in the crystal structures of spPfaD and spFabK, supporting a conserved role in cofactor stabilization.

Other water-mediated contacts in the TIM-barrel subdomain include G1633 and I1816, which is conserved in PKS-ERs, FabK, and PfaD but substituted by valine in scERc (Fig. S2). Three additional residues from the lid subdomain also participate in these interactions: V1834, V1838, and A1989 (Fig. 6B).

The conserved M1608 from motif I and W1985, located in the lid subdomain form hydrophobic interactions (Fig. 6B). W1985 is highly conserved in ERb, ERc, and PfaD. In PKS-ERs, this residue is replaced by histidine or tyrosine, whereas methionine is found in FabK (Fig. S2). In tmFabK crystal structure (PDB 5GVH; RMSD 1.41 Å), M276 forms equivalent hydrophobic contacts; its mutation to alanine abolishes FMN binding, suggesting a role in catalysis and in substrate positioning (Ha et al., 2017).

These interactions highlight a conserved FMN-binding environment. The effects of this environment on substrate binding and electron transfer were further explored through docking studies with NAD^+^, ACP, crotonyl-CoA, and the TUI inhibitor.

#### 2.6.2 NAD^+^

As mentioned above, TIM-barrel ER domains use NADH or NADPH as electron donors during catalysis. Crystallization trials of scERbc with these reduced cofactors were unsuccessful. Therefore, cofactor binding was analyzed by superimposing scERbc onto the soPfaD-NAD^+^ complex (PDB 4Z9R; RMSD 1.322 Å), as no structures of homologous TIM-barrel ER domains bound to NADH or NADPH were available at the time of analysis.

Ligand-binding interactions were initially analyzed in the soPfaD–NAD^+^ complex using PISA (Krissinel and Henrick, 2007) and PLIP (Schake et al., 2025), yielding a detailed interaction map of hydrogen bonds, hydrophobic contacts, and π-stacking interactions. These interaction patterns subsequently served as a reference to infer potential interactions in scERbc through structural alignments. NAD^+^ appears to occupy the same channel as FMN (Fig. 6C).

Docking analyses suggest that in scERb, the catalytic H1776 and the conserved S1968 residue, which is shared across PfaD, other ERb, and ERc homologs, could form hydrogen bonds with NAD^+^. In soPfaD, D369 forms an additional hydrogen bond; this residue is conserved in most ER domains but not in FabK. In the scERbc crystal structure, the corresponding residues D1870 (scERb) and D1316 (scERc) exhibit weak electron density and likely participate in similar interactions. However, N111 in soPfaD also forms a hydrogen bond with NAD^+^, whereas this position is occupied by K1610 in scERb (Fig. 6C).

Consistent with previous results in soPfaD, NAD^+^ may also engage in π-stacking interactions with H1776, W1985, and F1934 in scERb, the latter of which is replaced by H433 in soPfaD. These residues undergo conformational changes in soPfaD, adopting alternative rotamers while preserving hydrophobic contacts with FMN. Notably, W1985 and its equivalent, W482 in apo-soPfaD, adopt a similar orientation and sterically clash with NAD^+^ (Fig. 6D).

In scERb, L1964 and S1965, which is replaced by glycine in PfaD and other ER homologs (Fig. S2), appear to form water bridges with NAD^+^ and similarly undergo conformational shifts in soPfaD upon cofactor binding (Fig. 6D).

Overall, these interactions suggest a broadly conserved pattern across ER domains, in agreement with the docking predictions.

### 2.7 scERbc interactions with scACP and acyl groups

#### 2.7.1 scACP

The primary function of ER domains is the reduction of double bonds in acyl groups bound to ACP domains (Massengo-Tiassé and Cronan, 2009). To date, no structures of a TIM-barrel ER domain in complex with ACP have been experimentally determined. The ERbc–ACP interaction was investigated using AlphaFold3 by submitting scERb, scERc, and the first ACP domain from *Schizochytrium* sp. (scACP; PfaA residues 1116–1200) as separate inputs. The resulting predicted complex was subsequently aligned with the scERbc crystal structure.

**Fig. 7:**
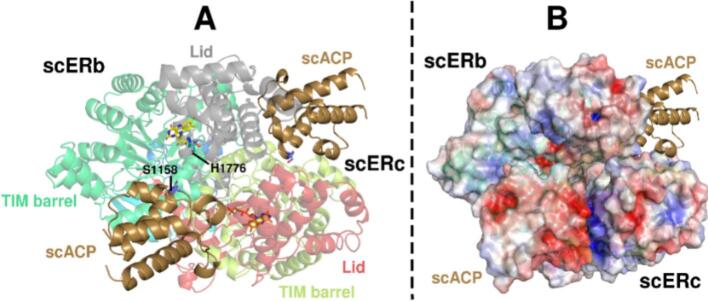
(A) Predicted docking of the first ACP domain (scACP; brown, PfaA residues 1116–1200) to scERbc, obtained with AlphaFold3 and aligned with the scERbc crystal structure (cartoon representation). scERb FMN is shown as yellow sticks, scERc FMN as orange sticks. The catalytic H1776 in scERb is shown in blue sticks, and the active S1158 of scACP is shown in light-blue sticks. (B) Electrostatic surface representation of scERbc and scACP, calculated with the Adaptive Poisson-Boltzmann Solver (APBS) in PyMOL. Colors range from blue (positive) to white to red (negative). The predicted interacting region of scERc shows a positively charged surface (blue), complementary to the negatively charged scACP. (For interpretation of the references to colour in this figure legend, the reader is referred to the web version of this article.)

In the predicted model, the interaction between scERb and scACP may involve the lid and TIM-barrel subdomains of scERc, and, to a lesser extent, the TIM-barrel subdomain of scERb. The active S1158 from ACP is oriented toward the catalytic H1776, and a similar pattern is observed for scERc (Fig. 7A).

As observed in other PUFA synthase systems (Santín et al., 2020), the ACP domain is highly electronegative. Positively charged regions in the lid and TIM-barrel subdomains of scERc likely contribute to electrostatic complementarity. The TIM-barrel subdomain of scERb may also participate in ACP binding, potentially serving as an anchoring point, although it is less electropositive. Together, these interactions could help stabilize the scACP docking to the ER domains (Fig. 7B).

#### 2.7.2 Crotonyl-CoA

To model the redox reaction involving the double bond, crotonyl-CoA was docked into the scERbc structure. This molecule represents an intermediate from the first round of modification during PUFA synthesis and thus constitutes the minimal unit containing a double bond. No TIM-barrel ER domain has been experimentally resolved in complex with crotonyl-CoA and attempts to co-crystallize the scERbc construct with this ligand were unsuccessful. Docking simulations were therefore performed using the SwissDock server (Bugnon et al., 2024; Eberhardt et al., 2021; Grosdidier et al., 2011; Trott and Olson, 2010), with the search space centered on the catalytic residue H1776 of scERb.

**Fig. 8:**
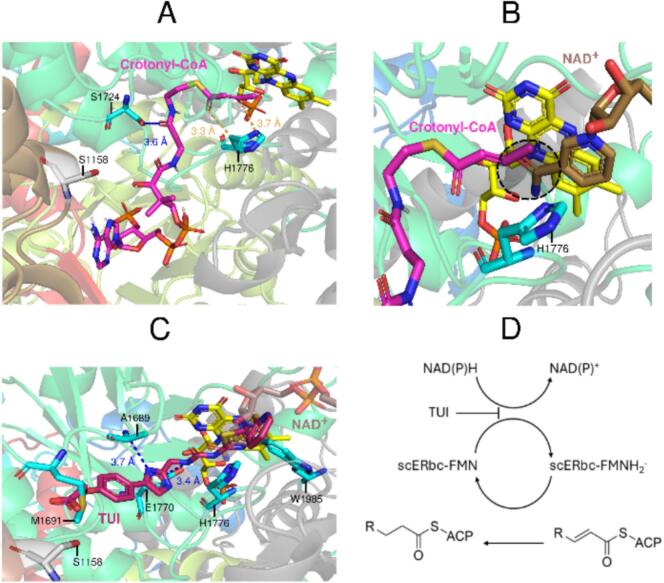
(A) Predicted interactions of crotonyl-CoA (magenta sticks) with scERbc (cartoon representation), generated using SwissDock (AutoDock Vina). The search space was centered around the catalytic H1776 from scERb. scERb residues are shown as cyan sticks and scACP active S1158 as gray sticks. Hydrogen bonds are shown in blue dashed lines, and potential Van der Waals interactions in light orange dashed lines. (B) Detailed close-up view of the predicted interactions between scERbc, crotonyl-CoA, and NAD^+^ (brown sticks). A steric clash is observed in the model (dashed circle). (C) Predicted binding mode of the TUI inhibitor (dark magenta sticks) in scERbc, obtained by superimposition of the spFabK-TUI crystal structure (PDB 2Z6J) onto scERbc. scERb residues are shown as cyan sticks and scACP active S1158 as gray sticks. NAD^+^ (brown sticks) is included to highlight the predicted steric clash between these molecules. Hydrogen bonds are shown in blue dashed lines. (D) Proposed reaction scheme for scERbc, inferred from docking and comparison with characterized enoyl reductases. The model is consistent with a two-step ping-pong mechanism. scERbc–FMN and scERbc–FMNH₂ denote oxidized and reduced states, respectively. TUI is shown as a predicted competitive inhibitor with respect to NAD(*P*)H. R represents an alkyl or alkenyl chain. (For interpretation of the references to colour in this figure legend, the reader is referred to the web version of this article.)

In the predicted models, crotonyl-CoA occupies a channel formed by the TIM-barrel and lid subdomains of scERb. The ligand appears to sit in a pocket between the active S1158 from ACP and H1776 (Fig. 8A). Potential hydrogen bonds are predicted for S1724, whose OG atom lies 3.6 Å from the carbonyl oxygen adjacent to the CoA thiol (Fig. 8A). S1724 is highly conserved across ER domains but is absent in FabK (Fig. S2).

Furthermore, the ND1 atom of H1776 lies 3.7 Å from the Cβ atom of crotonyl-CoA, consistent with a putative van der Waals contact. In addition, the carbonyl carbon of the crotonyl group is positioned approximately 3.3 Å from the Cβ of H1776, also suggesting a potential van der Waals interaction. Notably, the Cα atom of crotonyl-CoA sterically overlaps with NAD^+^, implying that the substrate and cofactor may not bind simultaneously (Fig. 8B).

### 2.8 TUI inhibitor

Further insight into the architecture and composition of the active site was obtained by modeling the interaction with TUI (2-(4-(2-((3-(5-(pyridin-2-ylthio)-thiazol-2-yl)ureido)methyl)-1H-imidazole-4-yl)phenoxy)acetic acid). This compound has been shown previously to bind competitively to NADPH and uncompetitively to crotonoyl-CoA in spFabK, thereby acting as an inhibitor (Saito et al., 2008). Accordingly, TUI was docked into the scERbc structure by superimposing the spFabK crystal structure in complex with TUI (PDB 2Z6J; RMSD 2.464 Å). In the resulting model, TUI occupies pockets overlapping with those of FMN, NAD^+^, and crotonyl-CoA. This suggests that TUI may sterically hinder cofactor and substrate binding, thereby potentially blocking catalysis (Fig. 8C).

Key residues predicted to interact with TUI include A1689 and E1770, which may form hydrogen bonds and are highly conserved across ER domains. Additionally, M1691 (replaced by leucine in PfaD), the catalytic H1776, and W1985 appear to sterically clash with the inhibitor (Fig. 8C). In the spFabK–TUI crystal structure, the catalytic H144 undergoes a conformational change (Saito et al., 2008), and W1985 may adopt a similar orientation similar to that observed in soPfaD upon NAD^+^ binding.

These interactions suggest that TUI may act as a competitive inhibitor with respect to NADH/NADPH and as an uncompetitive inhibitor with respect to crotonyl-CoA in scERbc, consistent with previous biochemical studies of spFabK. Although these observations require experimental validation, they are consistent with a sequential ping-pong catalytic mechanism proposed for related ER domains (Fig. 8D) (Saito et al., 2008; Zhang et al., 2021).

## 3 Discussion

Enoyl-ACP reductase (ER) domains catalyze the final reduction step in fatty acyl chain modification, converting unsaturated intermediates bound to ACP, such as crotonyl-ACP, into saturated acyl-ACP via FMN-dependent hydride transfer. Although ERs display extensive structural diversity, PUFA synthase ERs, FabK from bacterial FAS II, and certain PKS ERs adopt a TIM-barrel fold that positions FMN and NADH/NADPH for sequential reduction (Bukhari et al., 2014; Massengo-Tiassé and Cronan, 2009; Zhang et al., 2021).

According to domain predictions, thraustochytrid PUFA synthases are unique in containing two TIM-barrel ER domains: ERb in PfaB and ERc in PfaC (Fig. 1). Phylogenetic analyses place both domains near gammaproteobacterial PfaD, a standalone ER enzyme. Thraustochytrid ERc domains form a compact and highly conserved subclade. However, ERb domains are more divergent, including a *Schizochytrium* sp. ATCC 9695 lineage that shows lower identity to both PfaD and other thraustochytrid ERbs. Notably, the haptophyte *E. huxleyi* putative PUFA synthase only contains one ER domain that clusters within the ERb branch, consistent with an ancestral relationship among eukaryotic PUFA-type ERs (Fig. 2). Although *E. huxleyi* possesses a distinct, mammalian FAS-like PUFA synthase architecture (Fig. 1), the placement of ehER within the ERb clade suggests retention of ancestral ERb features prior to domain rearrangement.

The C-terminal region of this *E. huxleyi* PUFA synthase contains a DH–DH–ER arrangement, likely reflecting the organization of the common ancestor of haptophytes and thraustochytrids. In the thraustochytrid lineage, a genomic inversion in the single polypeptide may have produced an ER–DH–DH fragment, corresponding to the organization of the C-terminal region of PfaB and the N-terminal of PfaC. A subsequent duplication of this ER domain could have generated the second ER (ERc) found in PfaC, while the original ER (ERb) was retained in PfaB. This scenario could explain why ERc appears as a more conserved and recent clade in the phylogenetic tree, whereas ERb is more divergent and basal.

In our biochemical assays, recombinant scERb expressed alone in *E. coli* was insoluble and lacked FMN, whereas scERc was soluble, bound FMN, and predominantly formed homodimers in solution. Importantly, co-expressed scERb and scERc assembled into a stable scERbc heterodimer (Fig. S5B). Thermal stability assays revealed comparable melting temperatures for scERc (Tm = 37.88 ± 2.02 °C, *n* = 12), and scERbc (Tm = 39.21 ± 1.18 °C, n = 12) (Fig. S6), indicating that scERb is not independently stable and requires scERc for proper folding and cofactor binding. SEC-MALS additionally showed a minor monomeric population of scERc, suggesting that homodimerization is not strictly required for stability.

These observations prompted us to consider the broader architectural organization of scPfaB. In addition to ERb, scPfaB contains a KS-CLF didomain and an AT domain (Fig. 1). KS-CLF assemblies have been described as obligate heterodimers in PKSs (Keatinge-Clay et al., 2004) and proposed to adopt an intramolecular dimeric arrangement in gammaproteobacterial PUFA synthases (Santín et al., 2020). In contrast, AT domains are generally considered monomeric catalytic units (Liew et al., 2012; Lofeudo et al., 2026). Together, these observations suggest that scPfaB may function predominantly as a monomer in vivo. Interface analyses of the scERbc crystal structure and the AlphaFold3-predicted scERc homodimer revealed broadly similar interaction profiles. These results suggest that both homo- and heterodimer formation may be energetically feasible under physiological conditions (Table 2). Notably, an AlphaFold3 model of the scERb homodimer also yielded interface energetics within the range observed for the scERbc and scERc interfaces. However, recombinant scERb was unstable and unable to bind FMN when expressed alone. Together, these findings indicate that favorable predicted interface energetics alone are insufficient to define a stable or physiologically relevant assembly state.

Several observations support the physiological relevance of the scERbc heterodimer. In particular, together with ERc, PfaC also contains two DH domains, which are dimeric in related systems (Fig. 1) (Akey et al., 2010; Keatinge-Clay, 2008; Maier et al., 2008) and may interact intramolecularly to form the DH-DH active dimer within scPfaC. In isolation, scERc readily forms stable homodimers, as supported by the biochemical and SEC-MALS analyses. However, within the multidomain architecture of the PUFA synthase, scERc homodimerization may not be required in vivo, because the essential DH-DH dimer would already be established intramolecularly within PfaC. Instead, the multidomain organization of the PUFA synthase may favor the formation of the scERbc heterodimer as a functionally relevant ER assembly in thraustochytrids. In this context, scERc likely provides the structural framework required for FMN binding, while stabilizing and promoting proper folding of scERb. Thus, the observed scERbc heterodimerization is unlikely to arise solely from crystal packing or from nonspecific associations driven by sequence similarity.

Comparison with the experimentally resolved PfaD and FabK dimers further highlights the distinctive nature of the scERbc and scERc interfaces. Although scERbc and the modeled scERc exhibit interface areas comparable to those of FabK dimers, they contain substantially fewer hydrophobic interactions, which may account for their less favorable ΔiG values. In contrast, PfaD proteins display smaller interface areas and contain fewer polar contacts. Yet, PfaDs still exhibit more favorable interface energetics than scERbc or scERc (Table 2). These observations suggest that the scERbc/scERc dimerization mode is structurally and energetically distinct from previously characterized TIM-barrel ER dimers. Notably, both scERbc and scERc were analyzed as isolated domains outside the native multidomain context of scPfaB and scPfaC. Additional interactions contributed by neighboring domains within the PUFA synthase may therefore influence interface stabilization and assembly specificity in vivo.

Beyond the unique features of dimerization, the catalytic architecture of scERbc remains broadly similar to previously characterized TIM-barrel ERs. An N-terminal subdomain of approximately 80 amino acids is present, as in soPfaD (PDB 4Z9R) and spPfaD (PDB 6LKC), and is conserved in the sequences of related PUFA synthase ER domains (Fig. S2). This subdomain appears to be unique to PUFA synthase ERs, and its functional role has not yet been characterized. The N-terminal region may serve as a site for interaction with other components of the PUFA megasynthase.

Cofactor and substrate modeling suggests that, as in other ER enzymes, NAD^+^ binds within the FMN channel, potentially inducing rearrangements in residues such as W1985, similar to observations in soPfaD (PDB 4Z9R). Docking predicts that crotonyl-CoA occupies a distinct pocket, positioned between the active S1158 (scACP) and the catalytic H1776 (scERb). In this orientation, the substrate places its double bond near the active histidine, consistent with its proposed role as a general acid/base (Zhang et al., 2021). These results support a ping-pong mechanism, in which NAD^+^ dissociates after FMN reduction, allowing substrate entry (Fig. 8C) (Saito et al., 2008; Zhang et al., 2021).

Docking of the TUI inhibitor in scERbc suggests that it overlaps with the binding sites of FMN, NAD^+^, and crotonyl-CoA, generating steric clashes with the catalytic histidine and potentially inducing conformational rearrangements. These observations are consistent with the competitive inhibition with NADH, and partial uncompetitive substrate behavior reported for related enzymes (Saito et al., 2008), suggesting a similar inhibitory mechanism in scERbc.

Previous genetic studies proposed distinct roles for ERb and ERc in PUFA versus short-chain fatty acid synthesis, but these interpretations lacked biochemical validation and may reflect indirect effects on the stability or assembly of the PUFA synthase (Ling et al., 2020; Shi et al., 2021). Our findings provide direct evidence that scERb alone is unstable and likely nonfunctional, highlighting the need to reinterpret phenotypes from gene deletion or overexpression experiments in the context of domain folding, cofactor binding, and heterodimer formation.

In summary, our work provides the first structural and biochemical framework for thraustochytrid PUFA synthase ER domains, demonstrates the formation and physiological relevance of the scERbc heterodimer, and proposes a mechanistic model consistent with TIM-barrel ERs. Future studies will be essential to fully elucidate and validate the catalytic logic of eukaryotic PUFA synthase ER modules.

## 4 Materials and methods

### 4.1 Phylogenetic Analysis

Phylogenetic analyses were performed using the online platform Phylogeny.fr (Dereeper et al., 2008). Sequences alignments were generated with ClustalW to optimize alignment of divergent sequences (Table S1). Ambiguously aligned regions were removed using Gblocks to retain only reliably aligned positions. Phylogenetic trees were inferred with MrBayes under the WAG amino acid substitution model with invariable sites and gamma-distributed rate variation across sites. Markov chain Monte Carlo analyses were run for 100,000 generations, with sampling performed every 10 generations. The first 500 sampled trees were discarded as burn-in. The resulting tree was visualized and rooted using TreeDyn.

### 4.2 Strains and culture conditions

*E. coli* strain DH5α was used for molecular cloning, whereas the strain BL21(DE3) was employed for recombinant protein expression. All bacterial cultures were grown in LB medium. When required, antibiotics were added at the following concentrations: 50 μg/mL kanamycin and/or 100 μg/mL ampicillin (Sigma-Aldrich). Solid media were prepared with 1.5% (*w*/*v*) agar.

### 4.3 DNA manipulation and plasmid construction

scERb and scERc were amplified from synthetic DNA fragments codon-optimized for *E. coli* (IDT), corresponding to *pfaB* (UniProt accession Q94FB7, residues 1538–2059) and *pfaC* (UniProt Q94FB6, residues 982–1502), respectively. The amplified products were cloned into the pMAL-c2x and pET-29c vectors. PCR amplification was performed using Phusion High-Fidelity DNA polymerase (Thermo-Fisher) and custom-designed oligonucleotides (Sigma-Aldrich). PCR products were purified using the GeneJET PCR purification Kit (Thermo-Fisher) and DNA concentrations were determined with a NanoDrop ND-1000 spectrophotometer (Thermo-Scientific). DNA fragments were assembled by Gibson cloning (Gibson et al., 2009). The resulting constructs were transformed into *E. coli* DH5α by heat shock at 42 °C for 45 s. Colony screening was carried out using NZYTaq II polymerase (NZYTech), and positive clones were confirmed by Sanger sequencing (Eurofins Genomics).

### 4.4 Protein expression and purification

Protein expression was induced at OD_600_ 0.6–0.8 with 0.5 mM IPTG, followed by incubation at 18 °C. Cells were subsequently harvested and stored at −80 °C until further use.

Cells expressing His-tagged proteins were lysed in buffer A (0.3 M NaCl, 50 mM Tris-HCl pH 7.5, 20 mM imidazole, 1 mM PMSF, 1 mM DTT), whereas those expressing MBP-tagged proteins were lysed in buffer 1 (0.3 M NaCl, 50 mM Tris-HCl pH 7.5, 1 mM PMSF, 1 mM EDTA, 1 mM DTT). Proteins were purified by affinity chromatography using HisTrap HP or MBPTrap columns (Cytiva) and eluted using linear gradients (buffer B, 500 mM imidazole for His-tagged proteins; buffer 2, 10 mM maltose for MBP-tagged proteins).

*E. coli* cells co-expressing scERb and scERc were lysed in buffer A, and the lysate was sequentially purified using HisTrap HP and MBPTrap columns (Cytiva), with elution performed using the corresponding linear gradients (buffer B, 500 mM imidazole; buffer 2, 10 mM maltose).

All proteins were subsequently purified by size-exclusion chromatography (SEC) on a Superdex 200 10/300 GL column in SEC buffer (0.15 M NaCl, 25 mM Tris-HCl pH 7.5, 1 mM EDTA, 1 mM DTT). Purified proteins were stored at −80 °C until further use.

### 4.5 SEC-MALS

Size-exclusion chromatography coupled to multi-angle light scattering (SEC-MALS) was performed at Centro de Investigaciones Biológicas Margarita Salas (CIB-CSIC, Madrid, Spain). Purified scERb and scERbc were diluted to 1 mg/mL in SEC buffer and analyzed in duplicate by injecting 100 μL per run at a flow rate of 0.50 mL/min. Light scattering was measured using a DAWN EOS instrument (690 nm laser, 23 °C) coupled to an Optilab rEX refractive index detector and a quasi-elastic light scattering (QELS) module. Data were processed using Astra v5.3.2 software (Wyatt Technology).

### 4.6 Thermal shift assay

Thermal shift assays were performed using a fluorescence-based dye-binding method to assess the thermal stability of scERb, scERc and scERbc. Protein samples were prepared at 5 μM in SEC buffer and mixed with SYPRO Orange at 5× final concentration (Sigma-Aldrich). Fluorescence measurements were carried out on a StepOne Real-Time PCR System (Applied Biosystems) applying a temperature ramp from 25 °C to 90 °C at a rate of 0.5 °C/min. Fluorescence signals were recorded continuously and melting temperatures (Tm) were determined from the first derivative of the unfolding curves. Experiments included two biological replicates for scERb, buffer, and FMN, and three for scERbc and scERc, with four technical replicates each. Mean ± SD values were calculated from the combined dataset.

### 4.7 Protein crystallization and structure determination

scERc was concentrated to 10.5 mg/mL and crystallized in 0.1 M MES pH 6.5 with 12% (w/v) polyethylene glycol (PEG) 20,000, yielding crystals overnight. scERbc was concentrated to 15 mg/mL and crystallized in 0.1 M MES pH 6.5, supplemented with 0.1 M ammonium sulfate and 20% (w/v) PEG monomethyl ether 5000, with crystals appearing within 4–5 days. Crystals were cryoprotected with glycerol (35% *v*/v for scERc and 10% v/v for scERbc) and X-ray diffraction data were collected at the ALBA Synchrotron (Barcelona, Spain) on the XALOC beamline. Data processing was performed with the CCP4 suite (Battye et al., 2011). Structures were solved by molecular replacement using an AlphaFold3-predicted model (Abramson et al., 2024) in MolRep (CCP4). Refinement was carried out in Phenix (Adams et al., 2002) and manual model building was performed in Coot (Emsley and Cowtan, 2004). The final model, refined at 2.2 Å, was deposited in the PDB under the accession code 9SDD. Structural figures were generated with PyMOL (Schrödinger, LLC.) and multiple-sequence alignments were produced with Clustal-Omega (Madeira et al., 2024) and rendered with ENDscript (Robert and Gouet, 2014).

### 4.8 scERbc molecular docking

The interaction between scERbc and scACP (PfaA residues 1116–1200) was modeled using AlphaFold3 in monomer mode by inputting the amino acid sequences of the proteins. The predicted structure of scERbc was subsequently replaced with the experimentally determined scERbc crystal structure, while the AlphaFold3 predicted model of ACP (residues 1116–1200 from *Schizochytrium* sp. PfaA) was retained.

Noncovalent interactions within the scERbc complex were analyzed using PISA (Krissinel and Henrick, 2007) and PLIP (Schake et al., 2025). Molecular docking of crotonyl-CoA was performed with SwissDock (AutoDock Vina) (Bugnon et al., 2024; Eberhardt et al., 2021; Grosdidier et al., 2011; Trott and Olson, 2010). Ligands were provided as SMILES strings, and the binding site was defined around the catalytic histidine residue (H1776 in scERb and H1222 in scERc).

## CRediT authorship contribution statement

**Nahuel Lofeudo:** Writing – review & editing, Writing – original draft, Visualization, Methodology, Investigation, Formal analysis, Data curation, Conceptualization. **Gabriel Moncalian:** Writing – review & editing, Supervision, Resources, Project administration, Funding acquisition, Conceptualization.

## Funding

Spanish Ministry of Science, Innovation and Universities. Grant: PID2021-122164NB-I00, TED2021-129278B-I00 and PID2024-161085NB-I00.

## Declaration of competing interest

The authors declare that they have no known competing financial interests or personal relationships that could have appeared to influence the work reported in this paper.

## Data Availability

Data will be made available on request.
