# Supplementary material for: Thraustochytrid PUFA synthase ER domains form a stable heterodimer
Source: J Struct Biol X. 2026 Jun 10;14:100150. doi: 10.1016/j.yjsbx.2026.100150 (PMC13311796; doi:10.1016/j.yjsbx.2026.100150)
Supplement: Supplementary file 1 — Supplementary material 1 [file mmc1.docx]

**Thraustochytrid PUFA synthase ER domains form a stable heterodimer**

Nahuel Lofeudo^a^, and Gabriel Moncalian^a,1^

**SUPPORTING INFORMATION**

Table of contents:

Figure S1_____________________2

Figure S2_____________________3

Figure S3_____________________4

Figure S4_____________________4

Figure S5_____________________5

Figure S6_____________________5

Figure S7_____________________5

Figure S8_____________________6

Figure S9_____________________6

Table S1______________________7

**
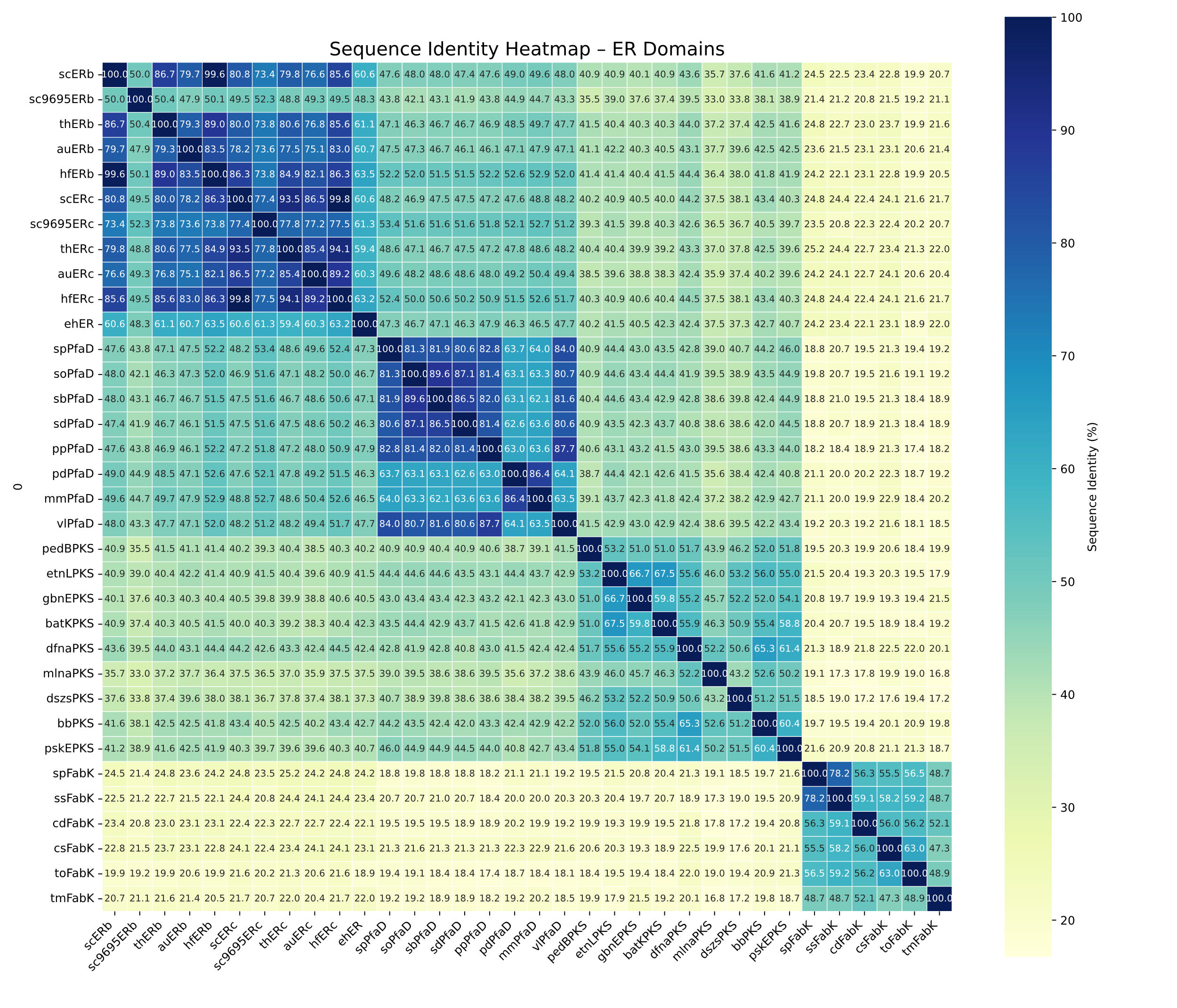
**

**Figure S1**. Heatmap of pairwise sequence identity (%) among selected ER domains. The UniProt accession numbers of the proteins used in the alignment are as follows: scERb, *Schizochytrium* sp. ATCC 20888 (Q94FB7); sc9695ERb, *Schizochytrium* sp. ATCC PTA-9695 (A0A1L6BQD4); thERb, *Thraustochytrium* sp. (A0A1B3PEI8); auERb, *Auriantiochytrium* sp. (A0A7H0U711); hfERb, *Hondaea fermentalgiana* (A0A2R5GGJ2); scERc, *Schizochytrium* sp. ATCC 20888 (Q94FB6); sc9695ERc, *Schizochytrium* sp. ATCC PTA-9695 (A0A1L6BQC8); thERc, *Thraustochytrium* sp. (A0A1B3PEI9); auERc, *Auriantiochytrium* sp. (A0A7H0U712); hfERc, *H. fermentalgiana* (A0A2R5GWM8); ehER, *Emiliania huxleyi* (R1EQS3); spPfaD, *Shewanella piezotolerans* (B8CQB6); soPfaD, *S. oneidensis* (Q8EGK4); sbPfaD, *S. baltica* (A0A0A7KUA3); sdPfaD, *S. denitrificans* (Q12KW7); ppPfaD, *Photobacterium profundum* (Q93CG5); pdPfaD, *Pseudoalteromonas denitrificans* (A0A1I1REH7); mmPfaD, *Moritella marina* (A0A5J6WHZ7); vlPfaD, *Vibrio lentus* (A0A2N7C6U5); pedBPKS, symbiont bacterium of *Paederus fuscipes* (Q6VT99); etnLPKS, *Sorangium cellulosum* (A9GJ18); gbnEPKS, *Burkholderia gladioli* (A0A4D8TUT6); batKPKS, *Pseudomonas fluorescens* (D4NZE6); dfnaPKS, *Bacillus velezensis* (A7Z6E3); mlnaPKS, *B. velezensis* (A7Z470); dszsPKS, *Sorangium cellulosum* (Q4U443); bbPKS, *Brevibacillus brevis* (C0ZGR0); pskEPKS, *Bacillus subtilis* (O34787); spFabK, *Streptococcus pneumoniae* (A0A0H2UNJ5); ssFabK, *Streptococcus salivarius* (A0A074IYA6); cdFabK, *Clostridioides difficile,* (A0A0H3N0T0); toFabK, *Terrisporobacter othiniensis* (A0A0B3WS17); tmFabK, *Thermotoga maritima* (Q9WZQ7).


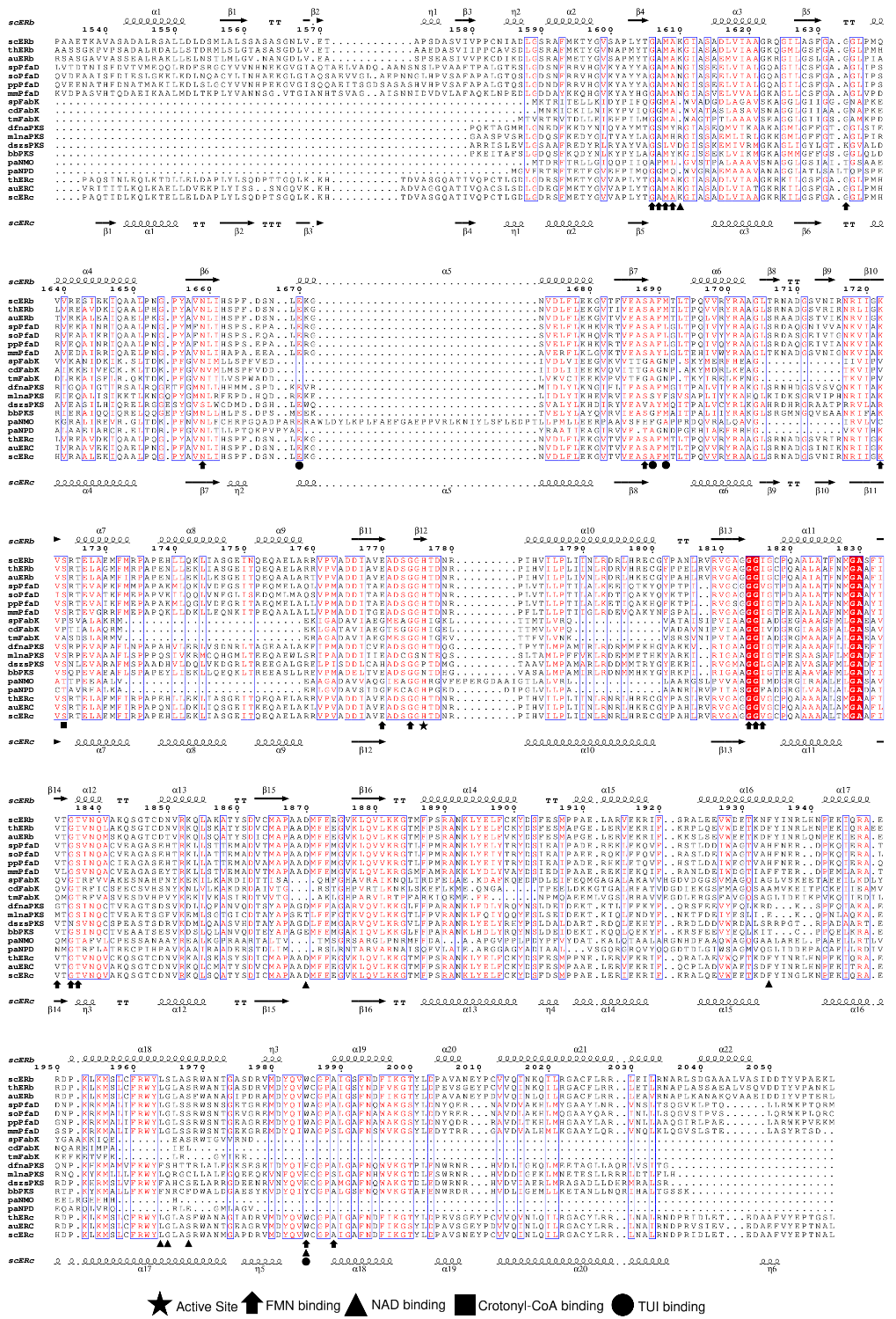


**Figure S2.** Structure-based multiple sequence alignment of ERbc. Numbering corresponds to ERb. Sequence homology is marked in red; sequence identity is presented with white letters on a red background. ERb and ERc secondary structure features (arrows for β-strands, and coils for α-helices) are indicated at the top and bottom, respectively. The UniProt accession numbers of the proteins used in the alignment are as follows: scERb, *Schizochytrium* sp. ATCC 20888 (Q94FB7); thERb, *Thraustochytrium* sp. (A0A1B3PEI8); auERb, *Auriantiochytrium* sp. (A0A7H0U711); spPfaD, *Shewanella piezotolerans* (B8CQB6); soPfaD, *S. oneidensis* (Q8EGK4); ppPfaD, *Photobacterium profundum* (Q93CG5); mmPfaD, *Moritella marina* (A0A5J6WHZ7); spFabK, *Streptococcus pneumoniae* (A0A0H2UNJ5); cdFabK, *Clostridioides difficile,* (A0A0H3N0T0); tmFabK, *Thermotoga maritima,* (Q9WZQ7); dfnaPKS, *Bacillus velezensis* (A7Z6E3); mlnaPKS, *B. velezensis* (A7Z470); dszsPKS, *Sorangium cellulosum* (Q4U443); bbPKS, *Brevibacillus brevis* (C0ZGR0); paNMO, *Pseudomonas aeruginosa*, (Q9HWH9); paNPD, *P. aeruginosa* (Q9I4V0); thERc, *Thraustochytrium* sp. (A0A1B3PEI9); auERc, *Auriantiochytrium* sp. (A0A7H0U712); scERc, *Schizochytrium* sp. ATCC 20888 (Q94FB6). The alignment was plotted with ENDscript.


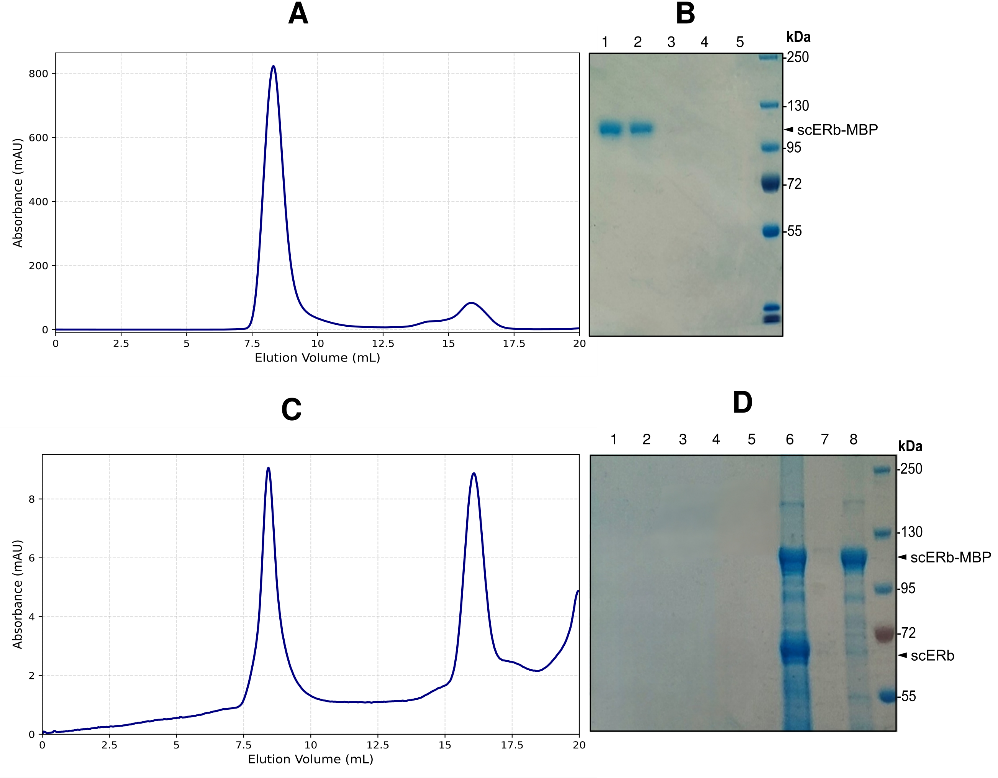


**Figure S3.** (A) Size-exclusion chromatography (SEC) elution profile of scERb. Absorbance at 280 nm is plotted as a function of elution volume (mL). (B) SDS-PAGE analysis (8% gel) of scERb. Lanes 1–2 show the protein fractions collected from the peak at 8.3 mL, and lanes 3–5 correspond to the peak at 15.87 mL. (C) SEC elution profile of scERb incubated with Factor Xa. (D) SDS-PAGE analysis (8% gel) of scERb after Factor Xa treatment. Lanes 1–2, fractions from peak at 8.3 mL; lanes 3–5, fractions from peak at 15.87 mL; lane 6, pellet after Factor Xa incubation; lane 7, soluble fraction after Factor Xa incubation; lane 8, scERb collected from affinity chromatography before Factor Xa treatment.


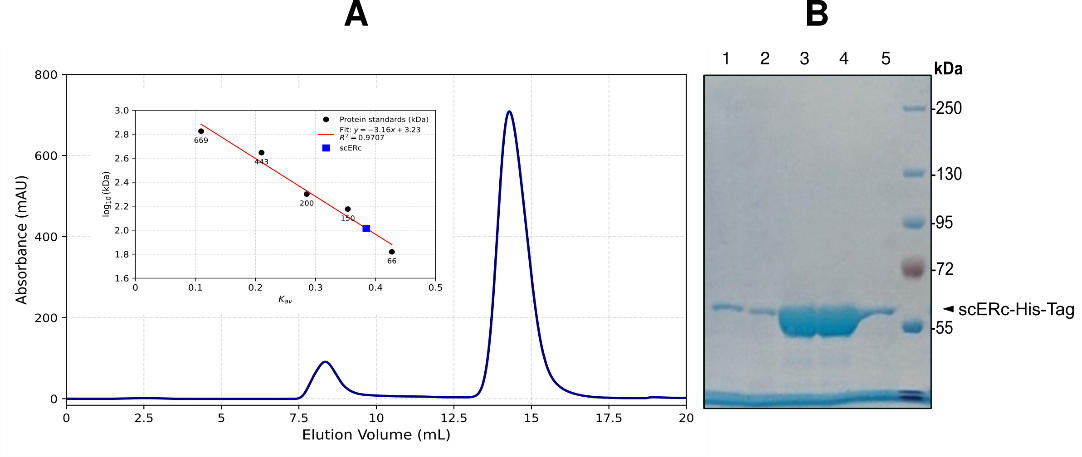


**Figure S4.** (A) Size-exclusion chromatography (SEC) elution profile of scERc and calibration curve. Absorbance at 280 nm is plotted as a function of elution volume (mL). (B) SDS-PAGE analysis (8% gel) of ERc. Lanes 1–2 show the protein fractions collected from the peak at 8.3 mL, and lanes 3–5 correspond to the peak at 14.29 mL.


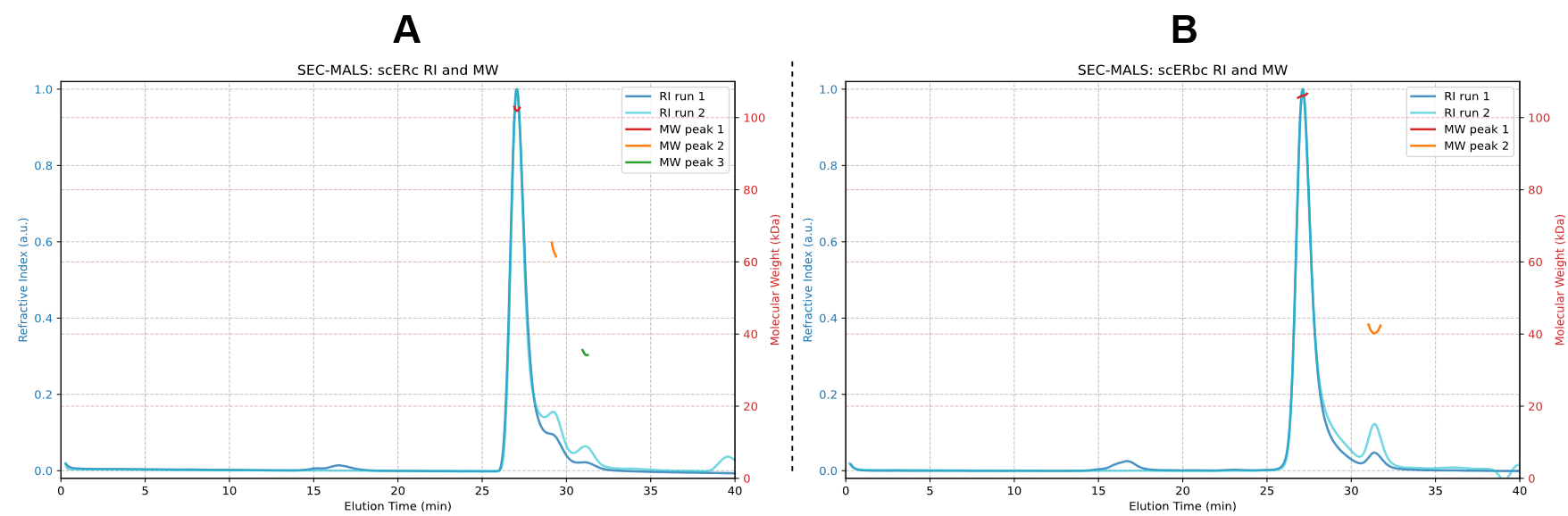


**Figure S5.** SEC-MALS analysis of (A) scERc and (B) scERbc. Refractive index (RI, blue) is shown for two independent runs (dark and light blue) on the left y-axis, while molecular weight (MW, right y-axis) is plotted for three elution peaks (red: peak 1, orange: peak 2, green: peak 3) in kDa. The RI trace indicates the protein elution profile, and the MW traces were derived from MALS measurements corresponding to the respective peaks.


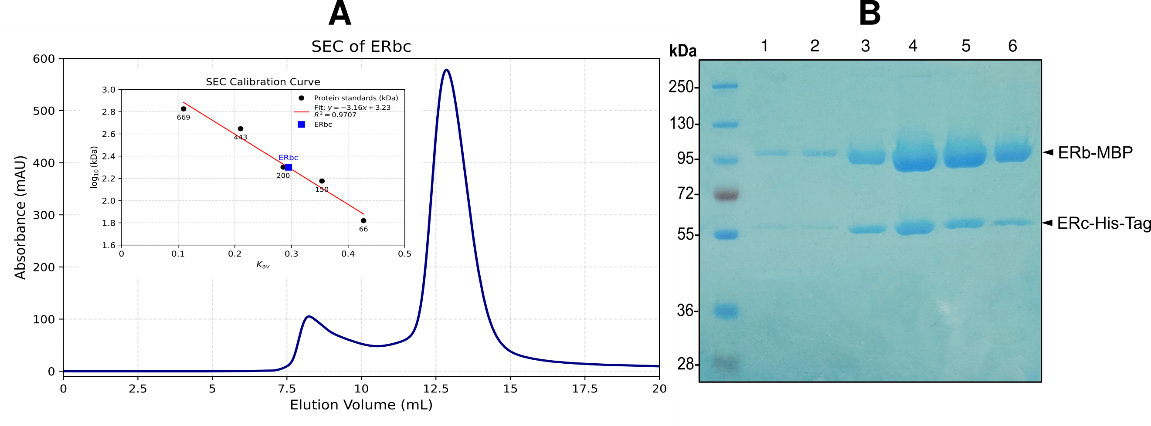


**Figure S6.** (A) Size-exclusion chromatography (SEC) elution profile of scERbc and calibration curve. Absorbance at 280 nm is plotted as a function of elution volume (mL). (B) SDS-PAGE analysis (8% gel) of ERbc. Lanes 1–2, fractions from peak at 8.3 mL; lanes 3–6, fractions from peak at 12.86 mL.


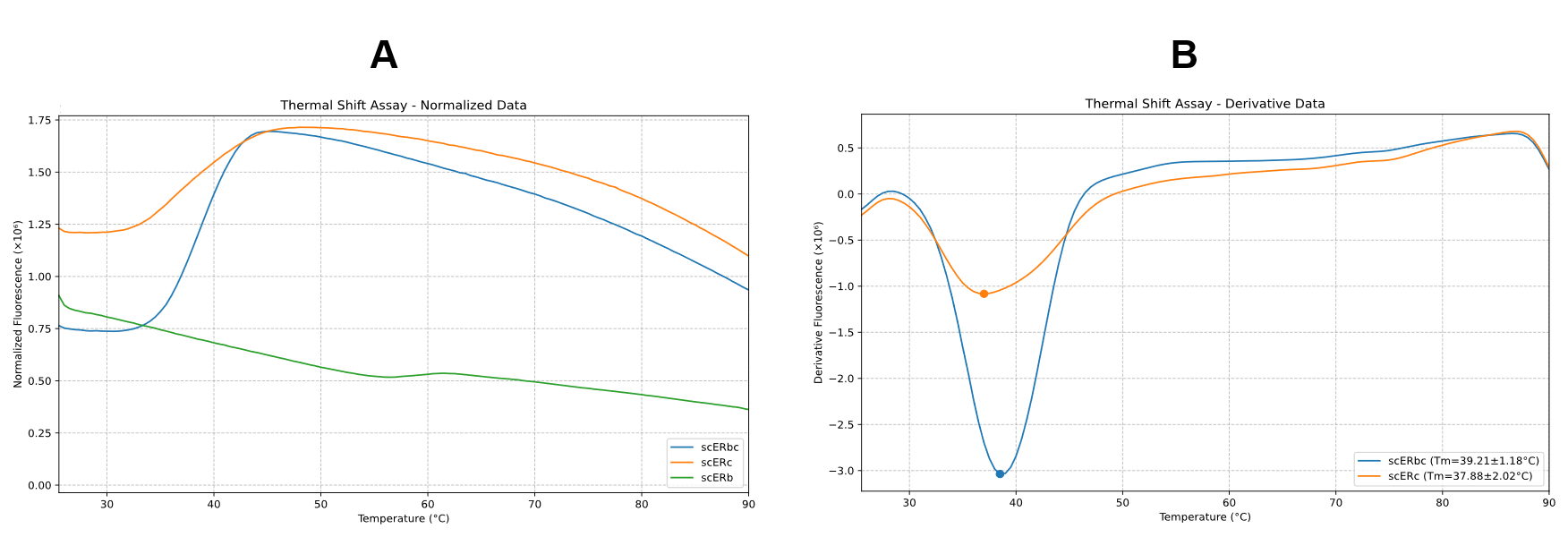


**Figure S7.** Thermal Shift Assay data. (A) Normalized fluorescence (×10⁶) for ERbc, scERb, scERc, FMN, and Buffer. (B) Derivative fluorescence (×10⁶) for ERbc, scERb, and scERc; melting temperatures (Tm) indicated as mean ± SD.


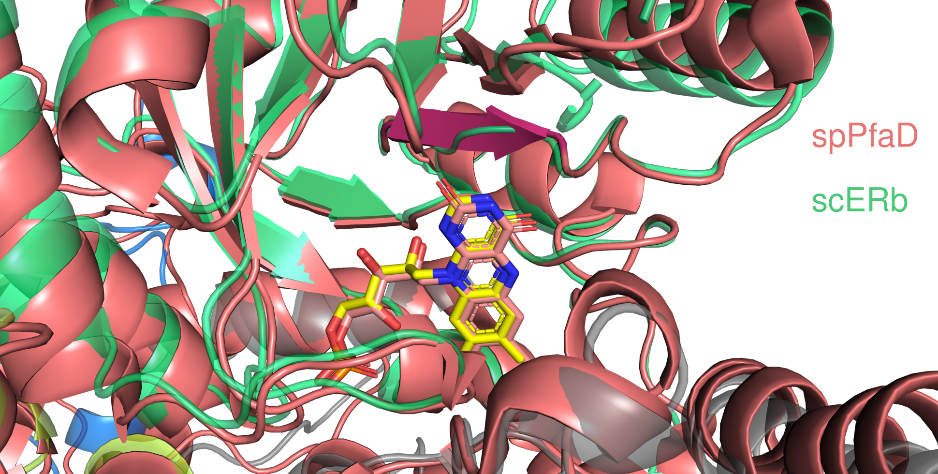


**Figure S8.** scERb (green) and spPfaD (salmon) structural alignment (cartoon representation). spPfaD, as other TIM barrel ER domains described possesses an extra β-strand (pink) that is absent in ERb and in ERc, in the TIM barrel subdomain proximal to the FMN cofactor.


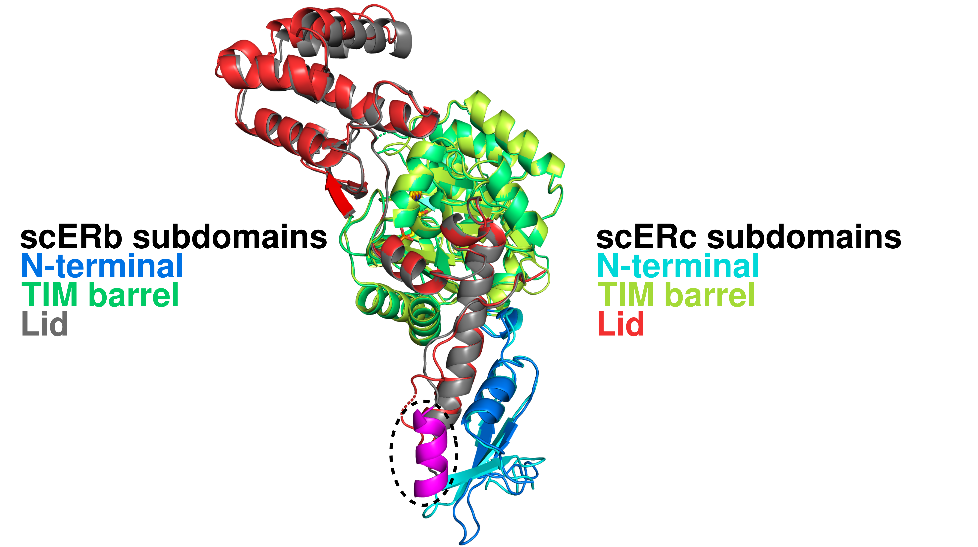


**Figure S9.** scERb and scERc structural alignment (cartoon representation). An extra α-helix (magenta) is observed in the lid subdomain of scERb.

**Table S1.** Protein sequences used for multiple sequence alignment and phylogenetic analyses.

| Protein name | UniProt entry |
| --- | --- |
| spFabK *Streptococcus pneumoniae* | A0A0H2UNJ5 |
| smFabK *Streptococcus mitis* | A0A081QKN0 |
| ssFabK *Streptococcus salivarius* | A0A074IYA6 |
| efFabK *Enterococcus faecalis* | A0A1B4XRD9 |
| csFabK *Clostridium sulfidigenes* | A0A084J8W2 |
| toFabK *Terrisporobacter othiniensis* | A0A0B3WS17 |
| cdFabK *Clostridioides difficile* | A0A0H3N0T0 |
| tmFabK *Thermotoga maritima* | Q9WZQ7 |
| woFabK *Weissella oryzae* | A0A069CWB8 |
| pedBPKS symbiont bacterium of *Paederus fuscipes* | Q6VT99 |
| etnLPKS *Sorangium cellulosum* | A9GJ18 |
| gbnEPKS *Burkholderia gladioli* | A0A4D8TUT6 |
| batKPKS *Pseudomonas fluorescens* | D4NZE6 |
| dszsPKS *Sorangium cellulosum* | Q4U443 |
| pskEPKS *Bacillus subtilis* | O34787 |
| mlnaPKS *Bacillus velezensis* | A7Z470 |
| bbPKS *Brevibacillus brevis* | C0ZGR0 |
| dfnaPKS *Bacillus velezensis* | A7Z6E3 |
| amPfaD *Aureispira marina* | A0A090A155 |
| pjPfaD *Psychroserpens jangbogonensis* | UPI00053EBE69 |
| ptPfaD *Psychroflexus torquis* | K4ICN9 |
| fpPfaD *Flammeovirga pacifica* | A0A1S1YZH8 |
| mcPfaD *Microcystis aeruginosa* | A0A0A1VV75 |
| fmPfaD *Fischerella major* | A0A1U7GVD1 |
| ttPfaD *Tolypothrix tenuis* | A0A1Z4N2P5 |
| nfPfaD *Nostoc flagelliforme* | A0A2K8SUN2 |
| soPfaD *Shewanella oneidensis* | Q8EGK4 |
| sbPfaD *Shewanella baltica* | A0A0A7KUA3 |
| sdPfaD *Shewanella denitrificans* | Q12KW7 |
| ppPfaD *Photobacterium profundum* | Q93CG5 |
| vgPfaD *Vibrio genomosp.* F10 | A0A1B9QWI7 |
| vlPfaD *Vibrio lentus* | A0A2N7C6U5 |
| vkPfaD *Vibrio kanaloae* | A0A4U1Z8B2 |
| spPfaD *Shewanella piezotolerans* | B8CQB6 |
| mmPfaD *Moritella marina* | A0A5J6WHZ7 |
| pdPfaD *Pseudoalteromonas denitrificans* | A0A1I1REH7 |
| cpPfaD *Colwellia psychrerythraea* | A0A099L1L5 |
| sc9695ERb *Schizochytrium* sp. ATCC PTA-9695 | A0A1L6BQD4 |
| ehER *Emiliania huxleyi* | R1EQS3 |
| sc9695ERc *Schizochytrium* sp. ATCC PTA-9695 | A0A1L6BQC8 |
| auERb *Auriantiochytrium* sp. | A0A7H0U711 |
| thERb *Thraustochytrium* sp. | A0A1B3PEI8 |
| scERb *Schizochytrium* sp. ATCC 20888 | Q94FB7 |
| hfERb *Hondaea fermentalgiana* | A0A2R5GGJ2 |
| auERc *Auriantiochytrium* sp. | A0A7H0U712 |
| thERc *Thraustochytrium* sp. | A0A1B3PEI9 |
| hfERc *Hondaea fermentalgiana* | A0A2R5GWM8 |
| scERc *Schizochytrium* sp. ATCC 20888 | Q94FB6 |
