# Supplementary material for: Thraustochytrid PUFA synthase ER domains form a stable heterodimer
Source: J Struct Biol X. 2026 Jun 10;14:100150. doi: 10.1016/j.yjsbx.2026.100150 (PMC13311796; doi:10.1016/j.yjsbx.2026.100150)
Supplement: Supplementary file 2 — Supplementary material 2 [file mmc2.pdf]

Full wwPDB X-ray Structure Validation Report (i)

Aug 19, 2025 – 01:00 pm BST

PDB ID	:	9SDD / pdb_00009sdd
Title	:	Crystal structure of ERb-ERc heterodimer from Schizochytrium sp.
Deposited on	:	2025-08-13
Resolution	:	2.20 Å(reported)

This wwPDB validation report is for manuscript review

This is a Full wwPDB X-ray Structure Validation Report.

This report is produced by the wwPDB biocuration pipeline after annotation of the structure.

We welcome your comments at *validation@mail.wwpdb.org*
A user guide is available at
https://www.wwpdb.org/validation/2017/XrayValidationReportHelp
with specific help available everywhere you see the (i) symbol.

The types of validation reports are described at
http://www.wwpdb.org/validation/2017/FAQs#types.

The following versions of software and data (see references (i)) were used in the production of this report:

MolProbity	:	4-5-2 with Phenix2.0rc1
Mogul	:	1.8.4, CSD as541be (2020)
Xtriage (Phenix)	:	2.0rc1
EDS	:	3.0
buster-report	:	1.1.7 (2018)
Percentile statistics	:	20231227.v01 (using entries in the PDB archive December 27th 2023)
CCP4	:	9.0.006 (Gargrove)
Density-Fitness	:	1.0.12
Ideal geometry (proteins)	:	Engh & Huber (2001)
Ideal geometry (DNA, RNA)	:	Parkinson et al. (1996)

For Manuscript Review

1 Overall quality at a glance (i)

The following experimental techniques were used to determine the structure:
X-RAY DIFFRACTION

The reported resolution of this entry is 2.20 Å.

Percentile scores (ranging between 0-100) for global validation metrics of the entry are shown in the following graphic. The table shows the number of entries on which the scores are based.

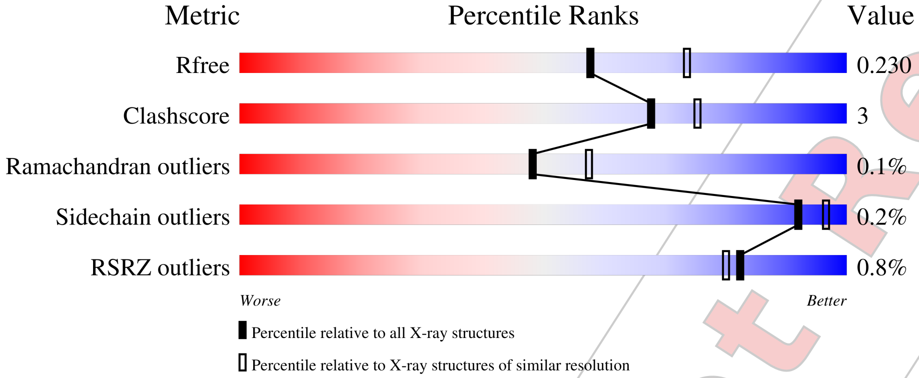

Metric	Whole archive (#Entries)	Similar resolution (#Entries, resolution range(Å))
R_{free}	164625	5791 (2.20-2.20)
Clashscore	180529	6634 (2.20-2.20)
Ramachandran outliers	177936	6560 (2.20-2.20)
Sidechain outliers	177891	6561 (2.20-2.20)
RSRZ outliers	164620	5791 (2.20-2.20)

The table below summarises the geometric issues observed across the polymeric chains and their fit to the electron density. The red, orange, yellow and green segments of the lower bar indicate the fraction of residues that contain outliers for >=3, 2, 1 and 0 types of geometric quality criteria respectively. A grey segment represents the fraction of residues that are not modelled. The numeric value for each fraction is indicated below the corresponding segment, with a dot representing fractions <=5% The upper red bar (where present) indicates the fraction of residues that have poor fit to the electron density. The numeric value is given above the bar.

Mol	Chain	Length	Quality of chain
1	B	527	92% 7% •
2	C	537	% / 87% 9% •

Validation Pipeline (wwPDB-VP) : 2.45.1

For Manuscript Review

2 Entry composition (i)

There are 6 unique types of molecules in this entry. The entry contains 8212 atoms, of which 0 are hydrogens and 0 are deuteriums.

In the tables below, the ZeroOcc column contains the number of atoms modelled with zero occupancy, the AltConf column contains the number of residues with at least one atom in alternate conformation and the Trace column contains the number of residues modelled with at most 2 atoms.

- Molecule 1 is a protein called Polyunsaturated fatty acid synthase subunit B.

Mol	Chain	Residues	Atoms	ZeroOcc	AltConf	Trace
1	B	521	Total 3967 C 2497 N 707 O 739 S 24	0	0	0

- Molecule 2 is a protein called Polyunsaturated fatty acid synthase subunit C.

Mol	Chain	Residues	Atoms	ZeroOcc	AltConf	Trace
2	C	520	Total 4008 C 2534 N 710 O 741 S 23	0	0	0

There are 16 discrepancies between the modelled and reference sequences:

Chain	Residue	Modelled	Actual	Comment	Reference
C	981	MET	-	initiating methionine	UNP Q94FB6
C	1503	VAL	-	expression tag	UNP Q94FB6
C	1504	ASP	-	expression tag	UNP Q94FB6
C	1505	LYS	-	expression tag	UNP Q94FB6
C	1506	LEU	-	expression tag	UNP Q94FB6
C	1507	ALA	-	expression tag	UNP Q94FB6
C	1508	ALA	-	expression tag	UNP Q94FB6
C	1509	ALA	-	expression tag	UNP Q94FB6
C	1510	LEU	-	expression tag	UNP Q94FB6
C	1511	GLU	-	expression tag	UNP Q94FB6
C	1512	HIS	-	expression tag	UNP Q94FB6
C	1513	HIS	-	expression tag	UNP Q94FB6
C	1514	HIS	-	expression tag	UNP Q94FB6
C	1515	HIS	-	expression tag	UNP Q94FB6
C	1516	HIS	-	expression tag	UNP Q94FB6
C	1517	HIS	-	expression tag	UNP Q94FB6

- Molecule 3 is FLAVIN MONONUCLEOTIDE (CCD ID: FMN) (formula: $C_{17}H_{21}N_4O_9P$) (labeled as "Ligand of Interest" by depositor).

For Manuscript Review

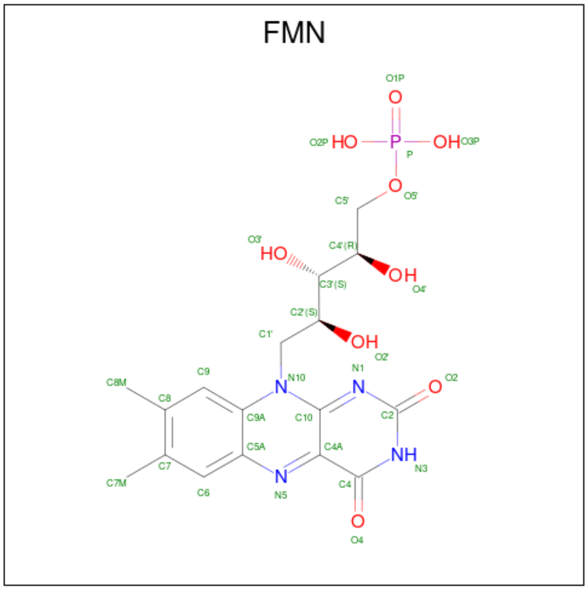

Mol	Chain	Residues	Atoms	ZeroOcc	AltConf
3	B	1	Total C N O P<br>31 17 4 9 1	0	0
3	C	1	Total C N O P<br>31 17 4 9 1	0	0

- Molecule 4 is SULFATE ION (CCD ID: SO4) (formula: O_4S).

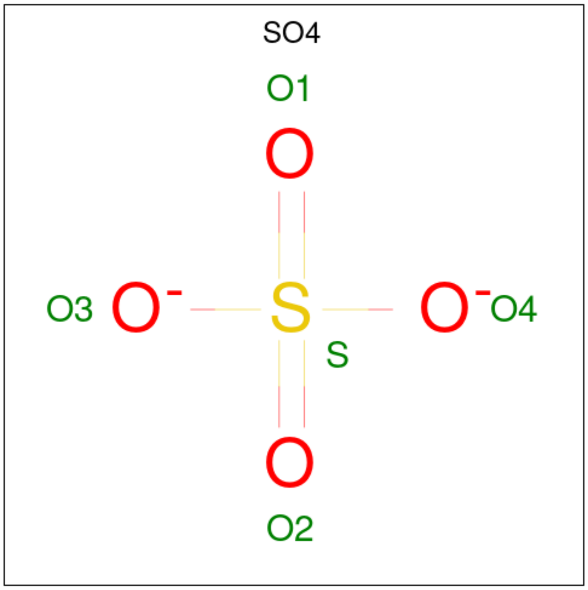

Mol	Chain	Residues	Atoms	ZeroOcc	AltConf
4	B	1	Total O S<br>5 4 1	0	0

- Molecule 5 is GLYCEROL (CCD ID: GOL) (formula: $C_3H_8O_3$).

For Manuscript Review

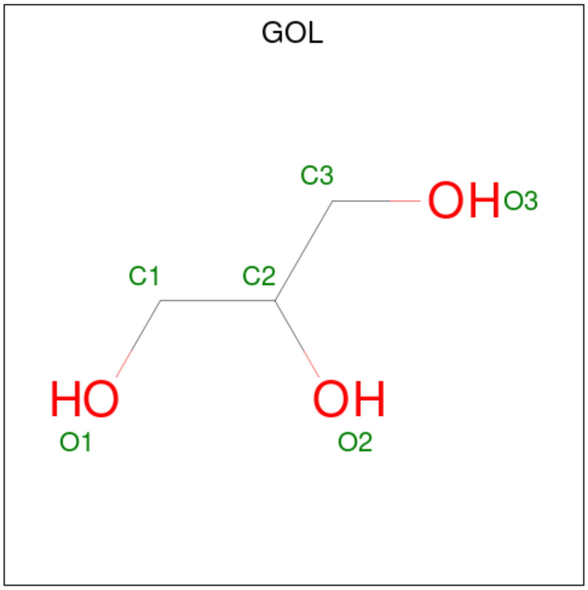

Mol	Chain	Residues	Atoms	ZeroOcc	AltConf
5	C	1	Total C O<br>6 3 3	0	0
5	C	1	Total C O<br>6 3 3	0	0
5	C	1	Total C O<br>6 3 3	0	0

- Molecule 6 is water.

Mol	Chain	Residues	Atoms	ZeroOcc	AltConf
6	B	89	Total O<br>89 89	0	0
6	C	63	Total O<br>63 63	0	0

For Manuscript Review

3 Residue-property plots ⓘ

These plots are drawn for all protein, RNA, DNA and oligosaccharide chains in the entry. The first graphic for a chain summarises the proportions of the various outlier classes displayed in the second graphic. The second graphic shows the sequence view annotated by issues in geometry and electron density. Residues are color-coded according to the number of geometric quality criteria for which they contain at least one outlier: green = 0, yellow = 1, orange = 2 and red = 3 or more. A red dot above a residue indicates a poor fit to the electron density (RSRZ > 2). Stretches of 2 or more consecutive residues without any outlier are shown as a green connector. Residues present in the sample, but not in the model, are shown in grey.

- Molecule 1: Polyunsaturated fatty acid synthase subunit B

Chain B:

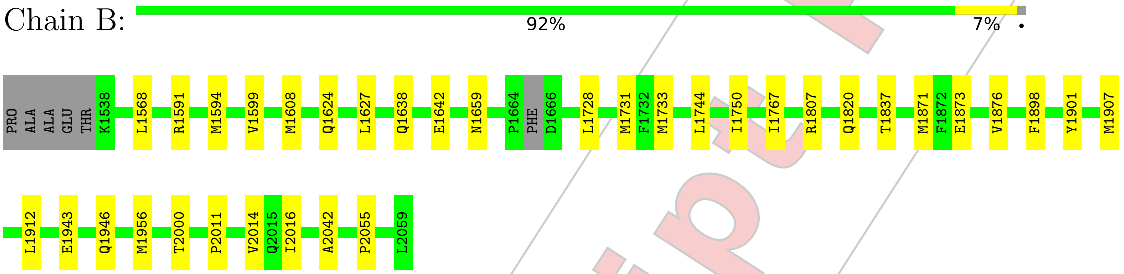

- Molecule 2: Polyunsaturated fatty acid synthase subunit C

Chain C:

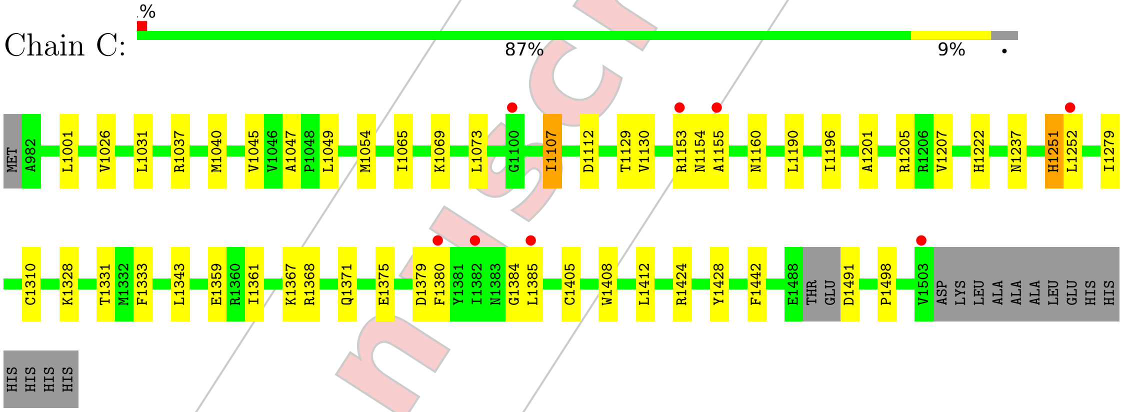

For Manuscript Review

4 Data and refinement statistics (i)

Property	Value	Source
Space group	I 1 2 1	Depositor
Cell constants<br>a, b, c, α, β, γ	117.97Å 79.11Å 119.45Å<br>90.00° 101.09° 90.00°	Depositor
Resolution (Å)	35.03 – 2.20<br>35.03 – 2.20	Depositor<br>EDS
% Data completeness<br>(in resolution range)	99.3 (35.03-2.20)<br>97.6 (35.03-2.20)	Depositor<br>EDS
R_{merge}	0.02	Depositor
R_{sym}	(Not available)	Depositor
$< I/\sigma(I) >$ [1]	3.02 (at 2.20Å)	Xtriage
Refinement program	PHENIX 1.21.2_5419	Depositor
R, R_{free}	0.185 , 0.230<br>0.185 , 0.230	Depositor<br>DCC
R_{free} test set	2609 reflections (4.75%)	wwPDB-VP
Wilson B-factor (Å^2)	44.5	Xtriage
Anisotropy	0.416	Xtriage
Bulk solvent k_{sol}(e/Å^3), B_{sol}(Å^2)	0.34 , 39.1	EDS
L-test for twinning[2]	$< \|L\| > = 0.49$, $< L^2 > = 0.32$	Xtriage
Estimated twinning fraction	0.009 for l,-k,h	Xtriage
F_o,F_c correlation	0.96	EDS
Total number of atoms	8212	wwPDB-VP
Average B, all atoms (Å^2)	56.0	wwPDB-VP

Xtriage's analysis on translational NCS is as follows: *The largest off-origin peak in the Patterson function is 4.98% of the height of the origin peak. No significant pseudotranslation is detected.*

[1] Intensities estimated from amplitudes.

[2] Theoretical values of $< |L| >$, $< L^2 >$ for acentric reflections are 0.5, 0.333 respectively for untwinned datasets, and 0.375, 0.2 for perfectly twinned datasets.

For Manuscript Review

5 Model quality (i)

5.1 Standard geometry (i)

Bond lengths and bond angles in the following residue types are not validated in this section: SO4, FMN, GOL

The Z score for a bond length (or angle) is the number of standard deviations the observed value is removed from the expected value. A bond length (or angle) with $|Z| > 5$ is considered an outlier worth inspection. RMSZ is the root-mean-square of all Z scores of the bond lengths (or angles).

Mol	Chain	Bond lengths		Bond angles	
		RMSZ	#$|Z|$ >5	RMSZ	#$|Z|$ >5
1	B	0.30	0/4037	0.46	0/5467
2	C	0.29	0/4085	0.47	0/5534
All	All	0.30	0/8122	0.46	0/11001

There are no bond length outliers.

There are no bond angle outliers.

There are no chirality outliers.

There are no planarity outliers.

5.2 Too-close contacts (i)

In the following table, the Non-H and H(model) columns list the number of non-hydrogen atoms and hydrogen atoms in the chain respectively. The H(added) column lists the number of hydrogen atoms added and optimized by MolProbity. The Clashes column lists the number of clashes within the asymmetric unit, whereas Symm-Clashes lists symmetry-related clashes.

Mol	Chain	Non-H	H(model)	H(added)	Clashes	Symm-Clashes
1	B	3967	0	4008	22	0
2	C	4008	0	4043	36	0
3	B	31	0	19	3	0
3	C	31	0	19	2	0
4	B	5	0	0	0	0
5	C	18	0	24	3	0
6	B	89	0	0	0	0
6	C	63	0	0	0	0
All	All	8212	0	8113	56	0

The all-atom clashscore is defined as the number of clashes found per 1000 atoms (including hydrogen atoms). The all-atom clashscore for this structure is 3.

For Manuscript Review

All (56) close contacts within the same asymmetric unit are listed below, sorted by their clash magnitude.

Atom-1	Atom-2	Interatomic distance (Å)	Clash overlap (Å)
2:C:1331:THR:HG22	2:C:1333:PHE:H	1.30	0.95
2:C:1343:LEU:HD13	2:C:1361:ILE:HD11	1.73	0.70
2:C:1153:ARG:HH12	2:C:1155:ALA:HA	1.62	0.65
1:B:1871:MET:HB2	1:B:1876:VAL:HB	1.79	0.64
2:C:1331:THR:HG21	2:C:1428:TYR:O	1.97	0.64
1:B:1907:MET:HE2	1:B:1912:LEU:HD12	1.81	0.62
2:C:1310:CYS:HB2	2:C:1328:LYS:HD3	1.84	0.59
2:C:1129:THR:HG21	5:C:1604:GOL:H2	1.83	0.59
2:C:1222:HIS:HB3	5:C:1602:GOL:H2	1.87	0.56
1:B:1943:GLU:O	1:B:1946:GLN:HG2	2.06	0.56
2:C:1107:ILE:HB	2:C:1112:ASP:HB2	1.89	0.55
2:C:1001:LEU:HB2	2:C:1026:VAL:HB	1.87	0.55
2:C:1129:THR:CG2	5:C:1604:GOL:H2	2.37	0.54
1:B:1591:ARG:HA	1:B:1594:MET:HE3	1.90	0.53
2:C:1129:THR:HG23	2:C:1130:VAL:HG23	1.90	0.53
2:C:1037:ARG:HA	2:C:1040:MET:HE2	1.90	0.52
2:C:1153:ARG:NH1	2:C:1155:ALA:HA	2.24	0.51
1:B:1901:TYR:O	1:B:1956:MET:HE1	2.10	0.51
1:B:1907:MET:HB3	1:B:1912:LEU:CD1	2.41	0.51
1:B:1820:GLN:HE22	2:C:1237:ASN:HD21	1.58	0.50
2:C:1045:VAL:HG11	2:C:1073:LEU:HB2	1.93	0.50
1:B:1624:GLN:HG3	1:B:2014:VAL:HG21	1.94	0.49
1:B:1638:GLN:O	1:B:1642:GLU:HG3	2.13	0.49
2:C:1207:VAL:HG12	2:C:1252:LEU:HD21	1.96	0.48
2:C:1031:LEU:HD23	2:C:1047:ALA:HB2	1.95	0.48
2:C:1054:MET:HA	3:C:1601:FMN:C5A	2.44	0.48
1:B:1568:LEU:HD13	1:B:2042:ALA:HB1	1.96	0.48
1:B:1733:MET:HE3	1:B:1767:ILE:HG13	1.94	0.48
2:C:1384:GLY:O	2:C:1385:LEU:HB2	2.14	0.47
2:C:1367:LYS:O	2:C:1368:ARG:HG3	2.14	0.47
1:B:1659:ASN:HD21	3:B:2101:FMN:HN3	1.63	0.47
1:B:1608:MET:HA	3:B:2101:FMN:N5	2.30	0.46
2:C:1190:LEU:HB3	2:C:1196:ILE:HG23	1.96	0.46
2:C:1251:HIS:O	2:C:1252:LEU:HB2	2.14	0.46
2:C:1343:LEU:CD1	2:C:1361:ILE:HD11	2.44	0.46
2:C:1371:GLN:O	2:C:1375:GLU:HB3	2.16	0.45
2:C:1359:GLU:H	2:C:1359:GLU:CD	2.24	0.45
2:C:1367:LYS:HD3	2:C:1424:ARG:NH1	2.32	0.45
1:B:1744:LEU:HB3	1:B:1750:ILE:HG12	2.00	0.44
2:C:1054:MET:HA	3:C:1601:FMN:N5	2.32	0.44

Continued on next page...

For Manuscript Review

Continued from previous page...

Atom-1	Atom-2	Interatomic distance (Å)	Clash overlap (Å)
1:B:1608:MET:HA	3:B:2101:FMN:C5A	2.48	0.44
1:B:1728:LEU:O	1:B:1731:MET:HG2	2.18	0.43
2:C:1412:LEU:HD23	2:C:1412:LEU:HA	1.86	0.43
2:C:1154:ASN:HB2	2:C:1160:ASN:OD1	2.19	0.43
2:C:1201:ALA:O	2:C:1205:ARG:HG3	2.19	0.43
2:C:1405:CYS:O	2:C:1408:TRP:HB3	2.20	0.42
1:B:1807:ARG:HH22	2:C:1491:ASP:HB2	1.85	0.42
1:B:1873:GLU:HG2	1:B:1898:PHE:CZ	2.55	0.41
1:B:2000:THR:HG21	1:B:2055:PRO:HD2	2.03	0.41
1:B:1599:VAL:HG11	1:B:1627:LEU:HB2	2.01	0.41
1:B:2011:PRO:HG2	1:B:2016:ILE:HD11	2.04	0.41
2:C:1065:ILE:HG22	2:C:1069:LYS:HE2	2.03	0.40
2:C:1442:PHE:CE1	2:C:1498:PRO:HD3	2.56	0.40
2:C:1379:ASP:O	2:C:1380:PHE:HB3	2.21	0.40
2:C:1049:LEU:HD12	2:C:1279:ILE:HD11	2.04	0.40
1:B:1744:LEU:HD23	1:B:1744:LEU:HA	1.95	0.40

There are no symmetry-related clashes.

5.3 Torsion angles (i)

5.3.1 Protein backbone (i)

In the following table, the Percentiles column shows the percent Ramachandran outliers of the chain as a percentile score with respect to all X-ray entries followed by that with respect to entries of similar resolution.

The Analysed column shows the number of residues for which the backbone conformation was analysed, and the total number of residues.

Mol	Chain	Analysed	Favoured	Allowed	Outliers	Percentiles	
1	B	517/527 (98%)	513 (99%)	4 (1%)	0	100	100
2	C	516/537 (96%)	505 (98%)	10 (2%)	1 (0%)	44	52
All	All	1033/1064 (97%)	1018 (98%)	14 (1%)	1 (0%)	48	57

All (1) Ramachandran outliers are listed below:

Mol	Chain	Res	Type
2	C	1251	HIS

For Manuscript Review

5.3.2 Protein sidechains (i)

In the following table, the Percentiles column shows the percent sidechain outliers of the chain as a percentile score with respect to all X-ray entries followed by that with respect to entries of similar resolution.

The Analysed column shows the number of residues for which the sidechain conformation was analysed, and the total number of residues.

Mol	Chain	Analysed	Rotameric	Outliers	Percentiles	
1	B	418/422 (99%)	417 (100%)	1 (0%)	92	96
2	C	422/436 (97%)	421 (100%)	1 (0%)	92	96
All	All	840/858 (98%)	838 (100%)	2 (0%)	92	96

All (2) residues with a non-rotameric sidechain are listed below:

Mol	Chain	Res	Type
1	B	1837	THR
2	C	1107	ILE

Sometimes sidechains can be flipped to improve hydrogen bonding and reduce clashes. All (11) such sidechains are listed below:

Mol	Chain	Res	Type
1	B	1624	GLN
1	B	1659	ASN
1	B	1739	HIS
1	B	1850	ASN
1	B	1937	ASN
2	C	983	GLN
2	C	1105	ASN
2	C	1237	ASN
2	C	1243	HIS
2	C	1286	GLN
2	C	1477	ASN

5.3.3 RNA (i)

There are no RNA molecules in this entry.

5.4 Non-standard residues in protein, DNA, RNA chains (i)

There are no non-standard protein/DNA/RNA residues in this entry.

For Manuscript Review

5.5 Carbohydrates (i)

There are no oligosaccharides in this entry.

5.6 Ligand geometry (i)

6 ligands are modelled in this entry.

In the following table, the Counts columns list the number of bonds (or angles) for which Mogul statistics could be retrieved, the number of bonds (or angles) that are observed in the model and the number of bonds (or angles) that are defined in the Chemical Component Dictionary. The Link column lists molecule types, if any, to which the group is linked. The Z score for a bond length (or angle) is the number of standard deviations the observed value is removed from the expected value. A bond length (or angle) with $|Z| > 2$ is considered an outlier worth inspection. RMSZ is the root-mean-square of all Z scores of the bond lengths (or angles).

Mol	Type	Chain	Res	Link	Bond lengths			Bond angles		
					Counts	RMSZ	$\#\|Z\|>2$	Counts	RMSZ	$\#\|Z\|>2$
3	FMN	C	1601	-	33,33,33	1.08	2 (6%)	48,50,50	1.38	7 (14%)
5	GOL	C	1604	-	5,5,5	0.38	0	5,5,5	0.55	0
5	GOL	C	1603	-	5,5,5	0.33	0	5,5,5	0.18	0
5	GOL	C	1602	-	5,5,5	0.22	0	5,5,5	0.38	0
4	SO4	B	2102	-	4,4,4	0.67	0	6,6,6	0.24	0
3	FMN	B	2101	-	33,33,33	1.08	2 (6%)	48,50,50	1.26	5 (10%)

In the following table, the Chirals column lists the number of chiral outliers, the number of chiral centers analysed, the number of these observed in the model and the number defined in the Chemical Component Dictionary. Similar counts are reported in the Torsion and Rings columns. '-' means no outliers of that kind were identified.

Mol	Type	Chain	Res	Link	Chirals	Torsions	Rings
3	FMN	C	1601	-	-	6/18/18/18	0/3/3/3
5	GOL	C	1604	-	-	2/4/4/4	-
5	GOL	C	1603	-	-	4/4/4/4	-
5	GOL	C	1602	-	-	2/4/4/4	-
3	FMN	B	2101	-	-	5/18/18/18	0/3/3/3

All (4) bond length outliers are listed below:

Mol	Chain	Res	Type	Atoms	Z	Observed(Å)	Ideal(Å)
3	B	2101	FMN	C4A-N5	4.14	1.38	1.30
3	C	1601	FMN	C4A-N5	3.81	1.38	1.30
3	B	2101	FMN	C10-N1	2.58	1.38	1.33

Continued on next page...

For Manuscript Review

Continued from previous page...

Mol	Chain	Res	Type	Atoms	Z	Observed(Å)	Ideal(Å)
3	C	1601	FMN	C10-N1	2.27	1.37	1.33

All (12) bond angle outliers are listed below:

Mol	Chain	Res	Type	Atoms	Z	Observed(^{o})	Ideal(^{o})
3	C	1601	FMN	C4-N3-C2	-3.61	118.97	125.64
3	C	1601	FMN	C4A-C10-N10	3.36	121.40	116.48
3	B	2101	FMN	C4-N3-C2	-3.12	119.87	125.64
3	C	1601	FMN	C10-C4A-N5	-3.02	118.44	124.86
3	B	2101	FMN	C4A-C10-N10	2.93	120.76	116.48
3	B	2101	FMN	C10-C4A-N5	-2.79	118.93	124.86
3	C	1601	FMN	C4A-C10-N1	-2.66	118.56	124.73
3	C	1601	FMN	C4A-C4-N3	2.58	119.73	113.19
3	B	2101	FMN	C4A-C4-N3	2.47	119.46	113.19
3	C	1601	FMN	C4-C4A-C10	2.33	120.71	116.79
3	B	2101	FMN	C4A-C10-N1	-2.22	119.58	124.73
3	C	1601	FMN	O4-C4-C4A	-2.02	121.24	126.60

There are no chirality outliers.

All (19) torsion outliers are listed below:

Mol	Chain	Res	Type	Atoms
3	C	1601	FMN	C5'-O5'-P-O1P
5	C	1604	GOL	C1-C2-C3-O3
5	C	1603	GOL	O1-C1-C2-C3
5	C	1603	GOL	C1-C2-C3-O3
5	C	1603	GOL	O1-C1-C2-O2
5	C	1604	GOL	O2-C2-C3-O3
3	B	2101	FMN	C2'-C3'-C4'-O4'
3	B	2101	FMN	C2'-C3'-C4'-C5'
3	C	1601	FMN	C4'-C5'-O5'-P
3	C	1601	FMN	C2'-C3'-C4'-O4'
5	C	1602	GOL	O2-C2-C3-O3
3	B	2101	FMN	C4'-C5'-O5'-P
3	B	2101	FMN	O3'-C3'-C4'-C5'
5	C	1603	GOL	O2-C2-C3-O3
3	B	2101	FMN	O3'-C3'-C4'-O4'
3	C	1601	FMN	C2'-C3'-C4'-C5'
3	C	1601	FMN	C5'-O5'-P-O2P
5	C	1602	GOL	C1-C2-C3-O3
3	C	1601	FMN	O3'-C3'-C4'-C5'

For Manuscript Review

There are no ring outliers.

4 monomers are involved in 8 short contacts:

Mol	Chain	Res	Type	Clashes	Symm-Clashes
3	C	1601	FMN	2	0
5	C	1604	GOL	2	0
5	C	1602	GOL	1	0
3	B	2101	FMN	3	0

The following is a two-dimensional graphical depiction of Mogul quality analysis of bond lengths, bond angles, torsion angles, and ring geometry for all instances of the Ligand of Interest. In addition, ligands with molecular weight > 250 and outliers as shown on the validation Tables will also be included. For torsion angles, if less then 5% of the Mogul distribution of torsion angles is within 10 degrees of the torsion angle in question, then that torsion angle is considered an outlier. Any bond that is central to one or more torsion angles identified as an outlier by Mogul will be highlighted in the graph. For rings, the root-mean-square deviation (RMSD) between the ring in question and similar rings identified by Mogul is calculated over all ring torsion angles. If the average RMSD is greater than 60 degrees and the minimal RMSD between the ring in question and any Mogul-identified rings is also greater than 60 degrees, then that ring is considered an outlier. The outliers are highlighted in purple. The color gray indicates Mogul did not find sufficient equivalents in the CSD to analyse the geometry.

For Manuscript Review

Ligand FMN C 1601

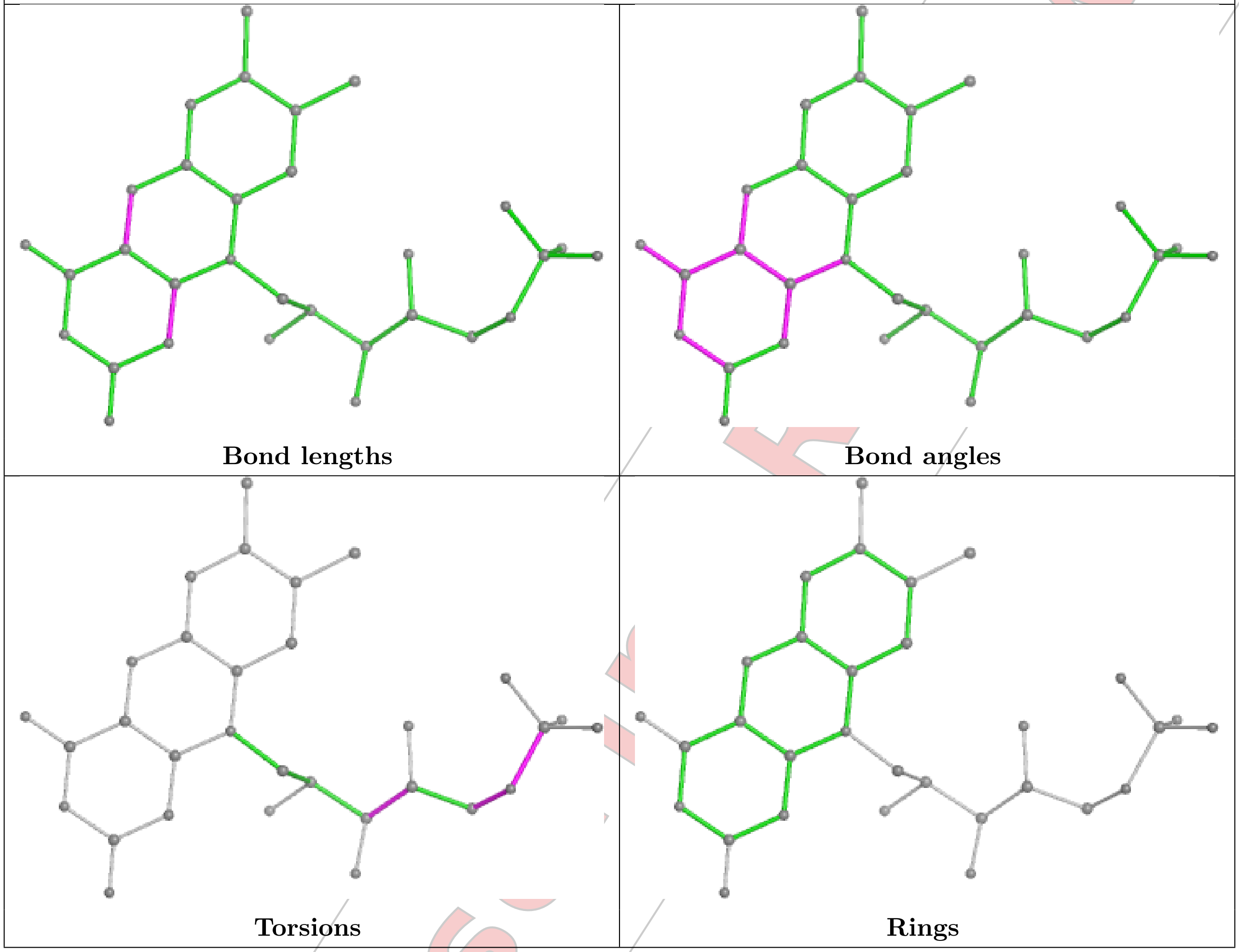

For Manuscript Review

Ligand FMN B 2101

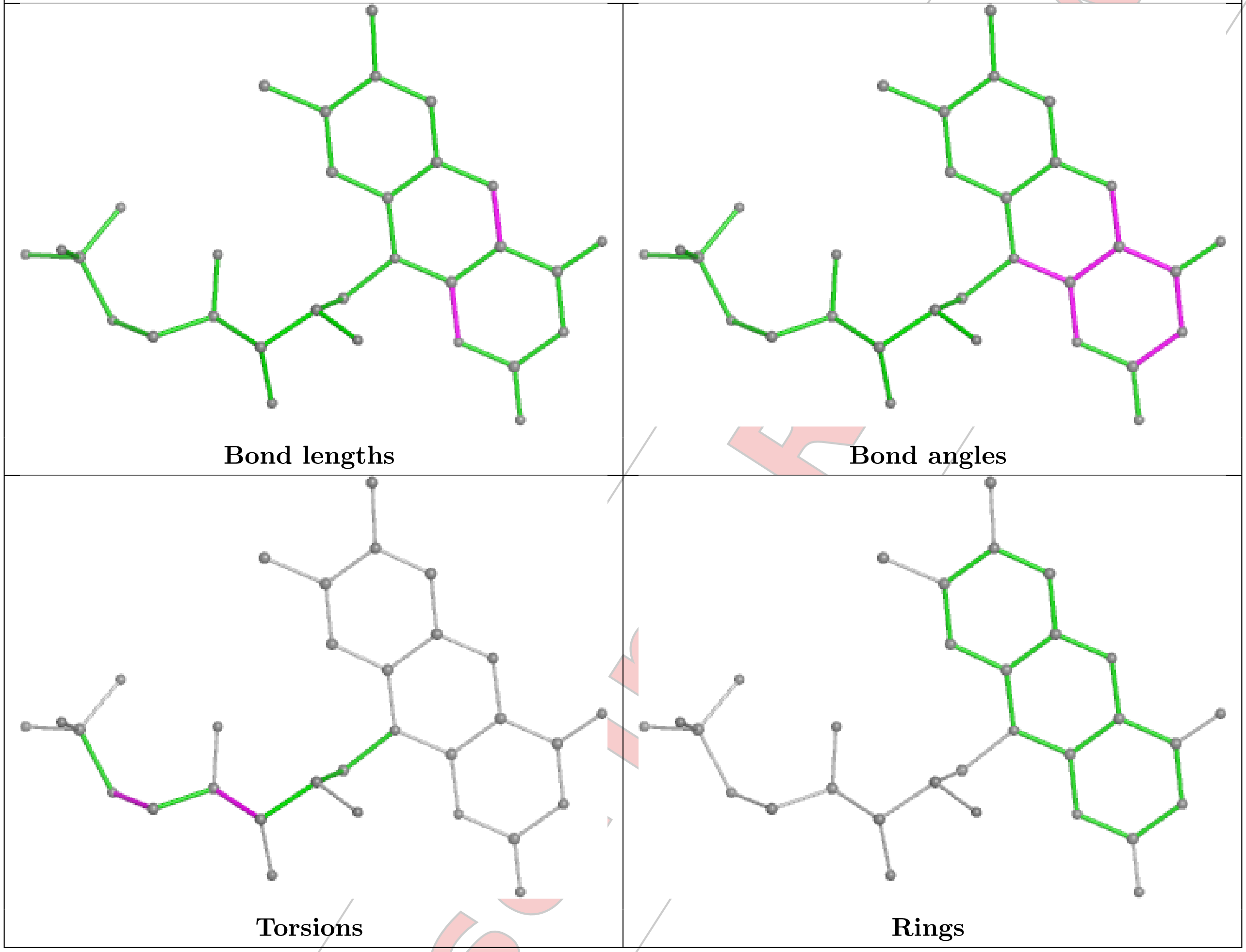

5.7 Other polymers (i)

There are no such residues in this entry.

5.8 Polymer linkage issues (i)

There are no chain breaks in this entry.

For Manuscript Review

6 Fit of model and data (i)

6.1 Protein, DNA and RNA chains (i)

In the following table, the column labelled '#RSRZ> 2' contains the number (and percentage) of RSRZ outliers, followed by percent RSRZ outliers for the chain as percentile scores relative to all X-ray entries and entries of similar resolution. The OWAB column contains the minimum, median, 95th percentile and maximum values of the occupancy-weighted average B-factor per residue. The column labelled 'Q< 0.9' lists the number of (and percentage) of residues with an average occupancy less than 0.9.

Mol	Chain	Analysed	<RSRZ>	#RSRZ>2			OWAB(Å^2)	Q<0.9
1	B	521/527 (98%)	-0.23	0	100	100	32, 51, 78, 95	0
2	C	520/537 (96%)	-0.05	8 (1%)	71	68	33, 58, 87, 103	0
All	All	1041/1064 (97%)	-0.14	8 (0%)	82	80	32, 54, 84, 103	0

All (8) RSRZ outliers are listed below:

Mol	Chain	Res	Type	RSRZ
2	C	1155	ALA	3.3
2	C	1100	GLY	2.9
2	C	1380	PHE	2.8
2	C	1385	LEU	2.6
2	C	1153	ARG	2.4
2	C	1382	ILE	2.3
2	C	1503	VAL	2.3
2	C	1252	LEU	2.1

6.2 Non-standard residues in protein, DNA, RNA chains (i)

There are no non-standard protein/DNA/RNA residues in this entry.

6.3 Carbohydrates (i)

There are no oligosaccharides in this entry.

6.4 Ligands (i)

In the following table, the Atoms column lists the number of modelled atoms in the group and the number defined in the chemical component dictionary. The B-factors column lists the minimum,

For Manuscript Review

median, 95th percentile and maximum values of B factors of atoms in the group. The column labelled ‘Q< 0.9’ lists the number of atoms with occupancy less than 0.9.

Mol	Type	Chain	Res	Atoms	RSCC	RSR	B-factors($Å^2$)	Q<0.9
5	GOL	C	1604	6/6	0.56	0.17	83,90,91,94	0
5	GOL	C	1602	6/6	0.74	0.17	66,70,71,73	0
5	GOL	C	1603	6/6	0.81	0.13	83,88,89,91	0
3	FMN	B	2101	31/31	0.97	0.06	32,37,41,42	0
3	FMN	C	1601	31/31	0.97	0.06	33,43,46,47	0
4	SO4	B	2102	5/5	0.97	0.08	47,53,58,58	0

The following is a graphical depiction of the model fit to experimental electron density of all instances of the Ligand of Interest. In addition, ligands with molecular weight > 250 and outliers as shown on the geometry validation Tables will also be included. Each fit is shown from different orientation to approximate a three-dimensional view.

Electron density around FMN B 2101:

$2mF_o$-DF_c (at 0.7 rmsd) in gray
mF_o-DF_c (at 3 rmsd) in purple (negative)
and green (positive)

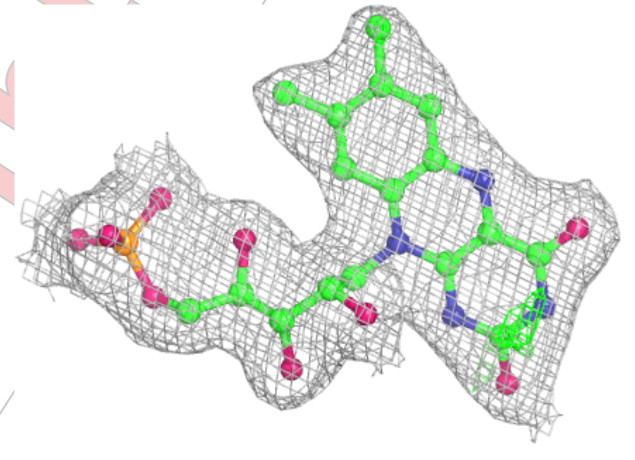

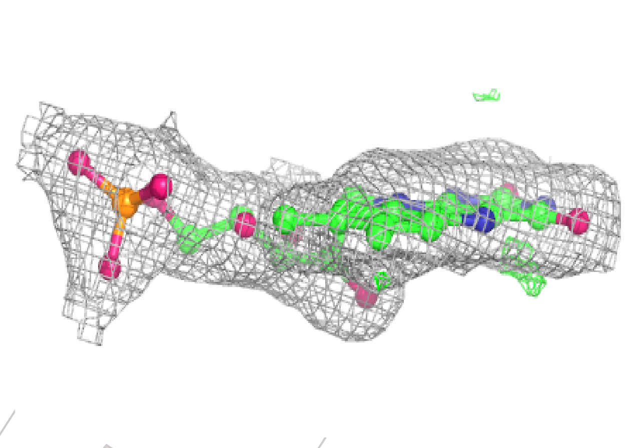

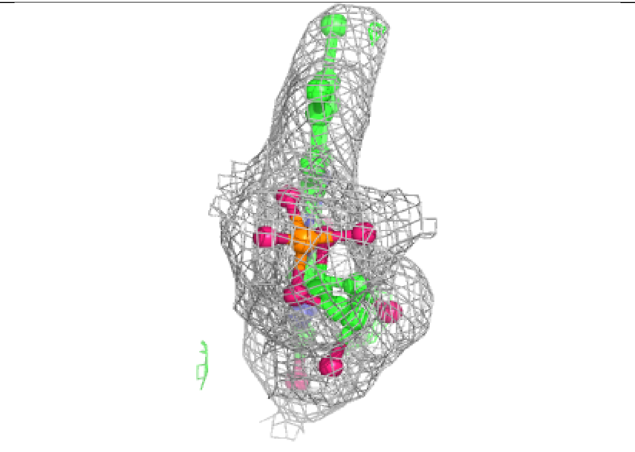

For Manuscript Review

Electron density around FMN C 1601:

$2mF_o$-DF_c (at 0.7 rmsd) in gray
mF_o-DF_c (at 3 rmsd) in purple (negative)
and green (positive)

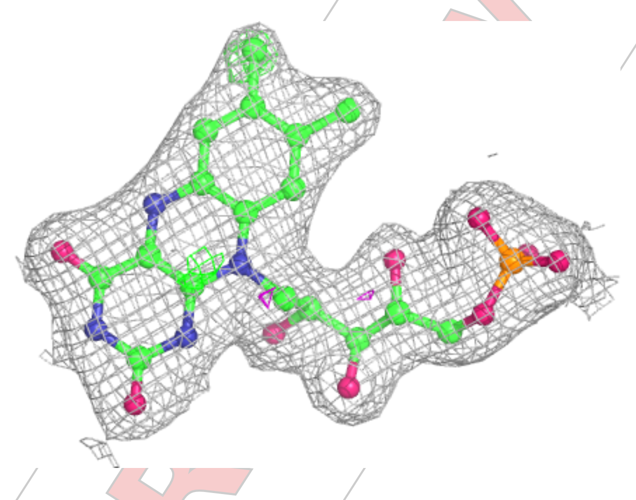

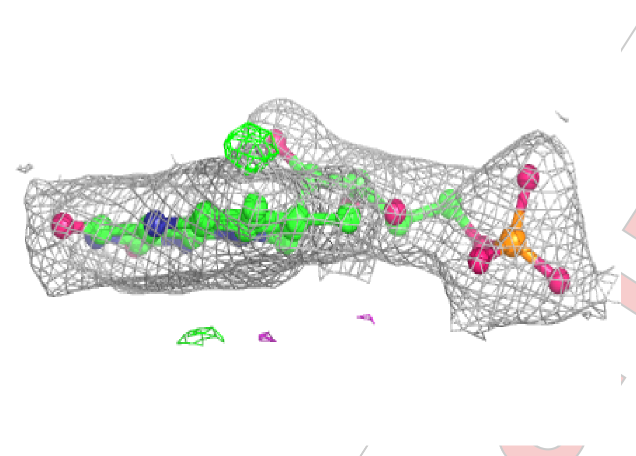

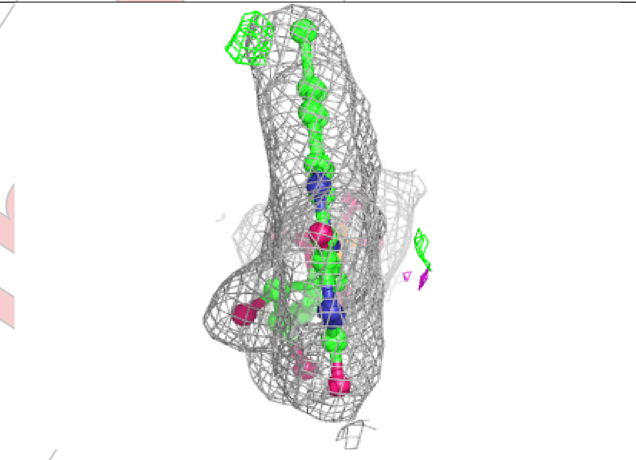

6.5 Other polymers ⓘ

There are no such residues in this entry.

For Manuscript Review
